# Deciphering the Leading-Edge Spatiotemporal Microenvironment of Hepatocellular Carcinoma for Targeted Drug Discovery Using SpaPred

**DOI:** 10.3390/ijms27156643

**Published:** 2026-07-25

**Authors:** Shibo Zhang, Ziqiao Li, Kexin Yu, Guang Shi, Yangguang Su, Xin Hu, Xiujie Chen

**Affiliations:** College of Bioinformatics Science and Technology, Harbin Medical University, Harbin 150081, China; 2023020558@hrbmu.edu.cn (S.Z.); louie19990528@163.com (Z.L.); ykx@hrbmu.edu.cn (K.Y.); 2025020438@hrbmu.edu.cn (G.S.); 2021020532@hrbmu.edu.cn (Y.S.); 2025020449@hrbmu.edu.cn (X.H.)

**Keywords:** hepatocellular carcinoma, tumor immune microenvironment, spatiotemporal transcriptomics analysis, deep learning, SpaPred model

## Abstract

The leading-edge (LE) of hepatocellular carcinoma (HCC) is a critical region driving malignant progression and is closely associated with high patient mortality and marked intratumoral heterogeneity. Multi-omics integration identified elevated expression of SPARC and IGFBP7 in the LE region, which was associated with stromal remodeling-related transcriptional programs and an immune-depleted microenvironment. Cell-cell communication and pathway analyses further suggested potential links between LE-associated stromal states and pro-invasive signaling programs. Furthermore, we developed SpaPred, which demonstrated favorable performance in inferring the spatiotemporal heterogeneity of HCC at the spatial resolution. This model overcomes the limitations of existing algorithms in analyzing the composition of tissue spatial structures. Finally, integration of in silico trajectory-perturbation and pharmacogenomic drug-response analyses prioritized Oxaliplatin, Belinostat, and Temsirolimus as candidate compounds associated with LE-related transcriptional programs. These drug predictions are computational and require experimental validation. Collectively, SpaPred provides a hypothesis-generating framework for investigating spatial heterogeneity and candidate therapeutic vulnerabilities in HCC.

## 1. Introduction

Complex intratumoral heterogeneity is the primary factor contributing to the low five-year relative survival rate of 22% among patients with hepatocellular carcinoma (HCC). This heterogeneity is not limited to genetic mutations in cancer cells but is also widespread in the dynamic interactions among different cell types within the tumor immune microenvironment (TIME) [1]. The leading-edge (LE) of tumor is regarded as an important region characterized by the expression of invasive and metastatic activities and presenting activated pro-tumor pathways including angiogenesis and epithelial-mesenchymal transition (EMT).

In recent years, with the emergence of single-cell technologies, researchers can unravel gene expression profiles and origins of biological functions within tumor tissues at the single-cell level, which helps to explore the mechanism of tumor heterogeneity [2,3]. Recent advances in spatial omics technologies have substantially improved our understanding of tissue organization, cellular interactions, and disease progression. Unlike conventional bulk transcriptomics and single-cell sequencing, spatial transcriptomics (ST) preserves the spatial coordinates of molecular measurements, enabling the investigation of gene expression patterns within their native tissue context. Spatial omics platforms, including 10× Genomics Visium, Slide-seq, MERFISH, and Stereo-seq, have been widely applied to characterize tumor ecosystems, immune infiltration landscapes, and cellular communication networks in various cancers. These technologies have revealed that tumor progression is driven not only by molecular alterations but also by highly dynamic spatial interactions among tumor cells, stromal cells, and immune populations.

Most studies have focused on explaining the origin and function of intratumoral heterogeneity in HCC, but few have paid attention to whether this heterogeneity shows regional specificity, leading to functional differences in different regions of the tumor [4,5]. Therefore, integrating single-cell spatiotemporal multi-omics technologies enables a systematic analysis of intratumoral heterogeneity and spatial structural characteristics at the cellular level to the tissue level, and reveals the molecular mechanisms of HCC progression in a multi-dimensional manner. In this study, we elucidated the cellular compositional heterogeneity of the TIME and its spatial distribution pattern and revealed the molecular basis and functional pathways of intratumoral heterogeneity that drive invasion and metastasis by regulating the microenvironment and cancer cell interactions [6,7,8].

To facilitate the interpretation of spatial omics data, several computational approaches have been developed. Cell2location employs a Bayesian probabilistic framework to integrate scRNA-seq reference datasets with spatial transcriptomics data, enabling the estimation of cell-type abundance in each spatial location. Similarly, RCTD (Robust Cell Type Decomposition) utilizes a statistical deconvolution model to infer cellular composition from mixed spatial transcriptomic spots using scRNA-seq references13. SPOTlight adopts a non-negative matrix factorization (NMF)-based framework to decompose spatial transcriptomic profiles into cell-type-specific proportions14. These methods primarily focus on cell-type deconvolution and have demonstrated strong performance in estimating cellular abundance across tissues. Beyond deconvolution, several spatial analysis frameworks have been proposed to investigate tissue architecture and spatial organization. Giotto provides a comprehensive toolbox for spatial data processing, visualization, clustering, neighborhood enrichment analysis, and cell-cell interaction inference15. Tangram introduces a machine learning-based mapping strategy that aligns single-cell transcriptomic profiles to spatial coordinates, thereby reconstructing spatial cellular distributions at higher resolution16. These methods have significantly advanced the analysis of spatial omics datasets and enabled the identification of biologically meaningful spatial patterns. However, several important limitations remain. First, most existing approaches are designed to estimate cell-type composition or map single-cell profiles onto spatial coordinates, rather than directly predicting spatiotemporal tumor architecture and dynamic tumor microenvironmental states. Second, these methods generally rely on matched single-cell reference datasets, which are often unavailable or subject to batch effects and sampling biases. Third, current frameworks mainly emphasize static spatial reconstruction and are less capable of characterizing the evolutionary trajectories and spatiotemporal heterogeneity associated with tumor progression. Finally, although these approaches effectively describe local cellular composition, they do not explicitly learn higher-order co-localization patterns between transcriptomic features and spatial contexts that may drive tumor evolution. To address these gaps, we developed SpaPred, a machine learning-based framework for predicting the spatiotemporal composition of HCC tissues. Unlike conventional deconvolution and mapping methods, SpaPred learns complex co-localization relationships between transcriptomic profiles and spatial coordinates to decode tumor architecture and TIME dynamics. Through feature representation learning and spatial pattern recognition, SpaPred captures spatiotemporal heterogeneity associated with HCC progression. Extensive 10-fold cross-validation and independent external validation demonstrated that SpaPred accurately predicts tumor architecture and TIME distribution patterns while exhibiting strong generalization capability. These characteristics position SpaPred as a robust computational framework for investigating tumor progression mechanisms and facilitating the development of precision intervention strategies. Benchmarking results against existing state-of-the-art spatial deconvolution algorithms demonstrate that SpaPred exhibits superior overall performance. More importantly, SpaPred possesses a unique dual-recognition capability: it can not only accurately infer cell type composition but also simultaneously resolve the intrinsic spatial structure of the tissue, whereas traditional methods typically focus on a single deconvolution task. Given the critical role of the LE region in tumor dissemination and microenvironmental remodeling, therapeutic intervention at this stage may offer a promising strategy for limiting HCC progression. While most current studies focus primarily on describing tumor spatial organization, there remains a need to translate spatiotemporal insights into actionable therapeutic opportunities. Therefore, beyond reconstructing the spatiotemporal landscape of HCC, we further investigated potential intervention strategies associated with disease progression, aiming to bridge spatial omics discoveries with precision oncology applications.

In this study, we systematically mapped the spatiotemporal heterogeneity landscape of HCC and identified the molecular mechanism by which the LE region establishes the immune-depletion microenvironment via stromal remodeling to promote tumor invasion and metastasis. Our findings offer new research directions to unveil TIME regulation mechanisms in the tumor LE region and to devise targeted precision therapeutics.

## 2. Result

### 2.1. Analysis of the Heterogeneity Composition of the Tumor Immune Microenvironment in Hepatocellular Carcinoma

**Figure 1:**
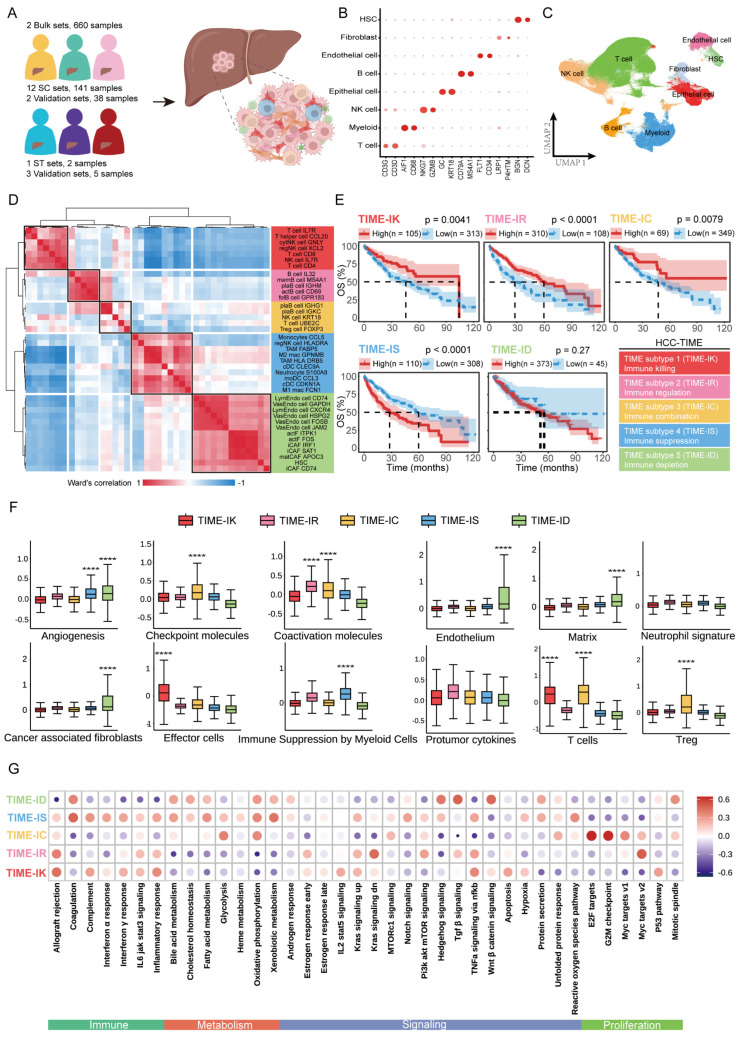
Cell annotation and classification of TIME subtypes in single-cell transcriptome samples. (**A**) Schematic representation of data collection and intratumor heterogeneity in primary hepatocellular carcinoma. (**B**) Specifically expressed genes for each cell type. (**C**) UMAP downscaled to show distribution of all major cell types. (**D**) Heatmap showing the five co-enrichment modules obtained from hierarchical clustering. (**E**) Survival analysis of patients in the TCGA-LIHC cohort based on five cellular modules. (**F**) Box plot represents the expression levels of each subset in the immunologic profiles obtained from the literature collection. (**G**) Heatmap of each cell module analyzed by GSVA functional enrichment.

To reveal the composition of the TIME of HCC, we performed scRNA-seq analysis on 141 patients with primary HCC (Figure 1A). After preprocessing, we retained and clustered 330,973 cells to obtain 13 cell clusters. These cell clusters were annotated as T cells, B cells, NK cells, myeloid cells, endothelial cells, fibroblasts, epithelial cells, and hepatic stellate cells (HSCs), based on known marker genes (Figure 1B,C and Figure S1C). K-means clustering analysis of copy number variation (CNV) revealed that epithelial cell cluster 6 contained both epithelial cells and reference cells. Its CNV score was also dramatically lower than that of other clusters (*p* < 0.001), suggesting it was a non-malignant cluster. The other epithelial cell clusters were discovered as malignant cell clusters (Figure S1D). To reveal cellular heterogeneity in TIME, we proceeded to deep subtyping annotation (Figure S1E). Given the complexity of the multi-cell interactions in the TIME, we applied the co-enrichment module analysis method. Based on hierarchical clustering, we grouped all the cells (except the epithelial cells) into five cell modules (CM1-CM5, Figure 1D). Based on the multi-index annotations of the TIME subtype, the following results were obtained: CM1 (TIME-IK, immune killing) is augmented by the majority of T and NK cells, expressing strongly over immunity-related functions (i.e., interferon, inflammatory response, and immune activation signal), significantly the marker of effector cell and T cell markers, thus evident immune killing features. CM2 (TIME-IR, immune regulation) is enriched with B cells for early immune activation/regulation, with highly co-activation molecular features and well-known immune regulatory roles, thus primarily playing antigen presentation and regulation of factors. CM3 (TIME-IC, immune combination) includes plasma cells, NK cells, regulatory T cells, etc. It actively expresses checkpoint molecules, co-stimulatory molecules, and regulatory T cell characteristics and strongly expresses various energy metabolism and circulatory regulatory functions. This reveals that the module composed of different immune cells works cooperatively in performing immune functions and realizing good immune effects [9]. CM1-3, as a subtype that significantly enhances immune function, is significantly associated with a favorable prognosis for the patients (Figure 1E–G). CM4 (TIME-IS, immune suppression) is a module constructed by many myeloid subtypes. All these cells communicate in ways that activate a pro-angiogenic and immunosuppressive myeloid cell cluster phenotype, together creating an immunosuppressive microenvironment associated with tumor growth [10]. Additionally, regulatory cells inhibit myeloid immune responses by the secretion of inhibitory cytokines and stimulating inflammatory responses between neutrophils and macrophages through IL-8 secretion, which regulates tumor suppression. In this way, the overall patient prognosis is further compromised [11]. CM5 (TIME-ID, immune depletion) is mainly enriched for endothelial cells and cancer-associated fibroblasts (CAFs) near tumor progression. These cells are in touch with mesenchymal cells to facilitate tumor proliferation and invasion and largely express TGF-β signaling and the Wnt/β-catenin signaling pathway, which is closely associated with malignant biological behavior of cancer [12,13]. Furthermore, CM4 and CM5 displayed immunosuppressive or immune-depleted transcriptional features. In the analyzed cohorts, higher TIME-ID scores showed a non-significant trend toward unfavorable prognosis; therefore, its independent prognostic relevance requires validation in larger cohorts (Figure 1E–G).

### 2.2. The Molecular Interactions and Heterogeneity Map of the Tumor Immune Microenvironment

**Figure 2:**
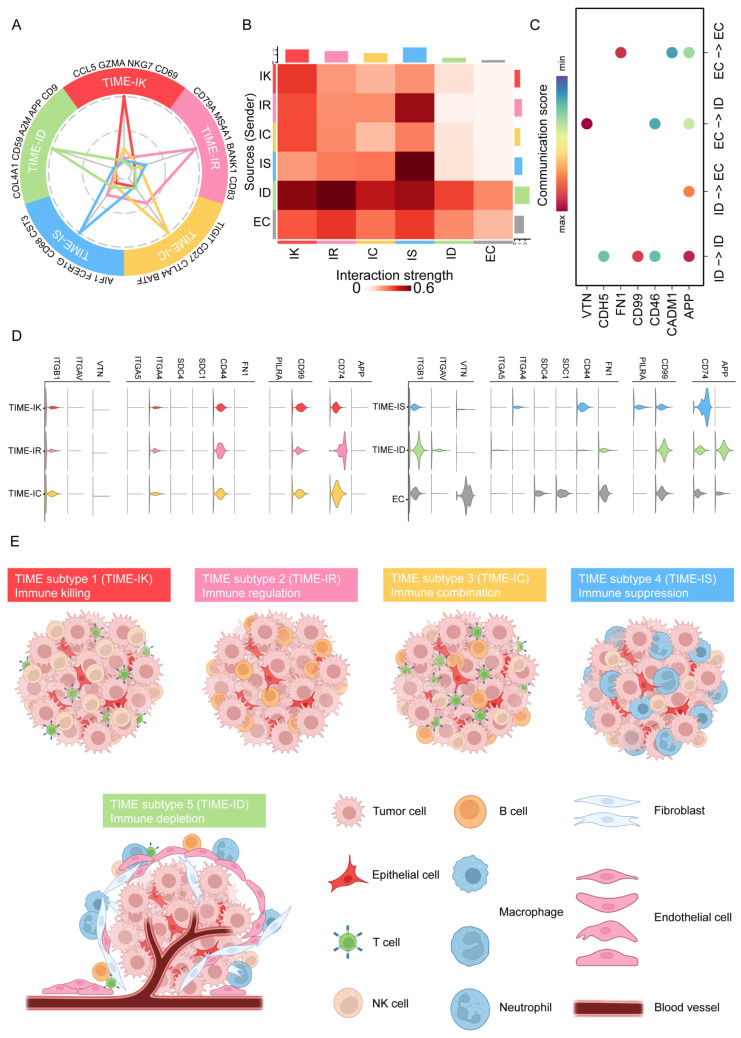
TIME subtype functional analysis and inter-subtype signaling. (**A**) Radar plot representing the level of specific marker enrichment for each TIME subtype. (**B**) Heatmap of signaling communication weights between TIME subtypes. (**C**) Bubble plot showing ligand-receptor expression for interactions between immune depletion subtype and malignant epithelial cells. (**D**) Violin plot showing gene expression levels associated with key signaling molecules. (**E**) Schematic representation of the TIME subtype containing individual cells in the immune microenvironment of hepatocellular carcinoma tumor.

The heterogeneous biological functions resulting from HCC intratumoral heterogeneity have been further explored through an intracellular signaling analysis, where sub-specific features of molecular interaction profiles and signaling pathways associated with subtypes can be identified. Associated heatmaps further illustrate some striking relationships between TIME subtypes and clinical information (age, TNM staging). Besides, the TIME-ID subtype is enrichment-related to advanced tumor status and aging (Figure S2A), suggesting its link with malignant transformation. Radar plots showed different and uncorrelated gene expression of TIME subtypes (Figure 2A). There was a complicated interaction between cancer cells and TIME subtypes. TIME-ID, as one of the key signal senders, mainly contributes to the critical molecular expression processes between tumor cells and local surroundings (Figure 2B). Further ligand-receptor analysis showed that TIME-ID activates the APP molecule, acting on tumor cells and their local surroundings. Cancer cells modulate their own intercellular adhesion ability and cell motility pathway by the CD99 signaling pathway, which causes the formation of a matrix that suppresses immunological function and stimulates cancer cell proliferation and invasion ability. The cancer cell controls VTN molecules to stimulate matrix remodeling and cytoskeletal pathways, forming a matrix barrier. Meanwhile, the activation of FN1 signaling decreases tumor cell adhesion, which reinforces their invasiveness. Finally, the convergent effect of multiple molecular signals results in the establishment of a depleted microenvironment and favors aggressive tumor growth (Figure 2C,D and Figure S2B). TIME-ID strongly induces pathways such as tissue migration, tumor LE, remodeling of the extracellular matrix, and cytoskeletal assembly to further construct a matrix barrier against immune cell infiltration into the tumor core and the weakening of the antitumor immune response. Meanwhile, TIME-ID strongly activates pro-tumor pathways such as PI3K-Akt, MAPK, and ECM-receptor interaction to synergistically promote malignant tumor development (Figure S2C). Because of the heterogeneity in TIME, TIME-ID disturbs the immune-response ability of TIME-IS by APP-CD74 pathway disruption, leading to the loss of its antigen-presenting function [14]. TIME-IR upregulates the CLEC2C signaling factor, which transmits signals with the NK cell marker KLRB1 in TIME-IK [15] to promote the activation of the NK cell and enhance its recognition and killing effect. We used two independent datasets (GSE151530, PMID: 34145435) to validate the robustness of TIME heterogeneity composition recognition, and the results show that the associated subtype has mutually high independence and maintains similar biological functions in the validation cohort, indicating the authenticity and robustness of our classification model (Figure S3). According to the cell composition and function features, we specifically summarize the distribution pattern of each type and depict the cellular environment in the tumor (Figure 2E).

### 2.3. Molecular Interactions and Functional Analysis of Spatial Structures in Hepatocellular Carcinoma

**Figure 3:**
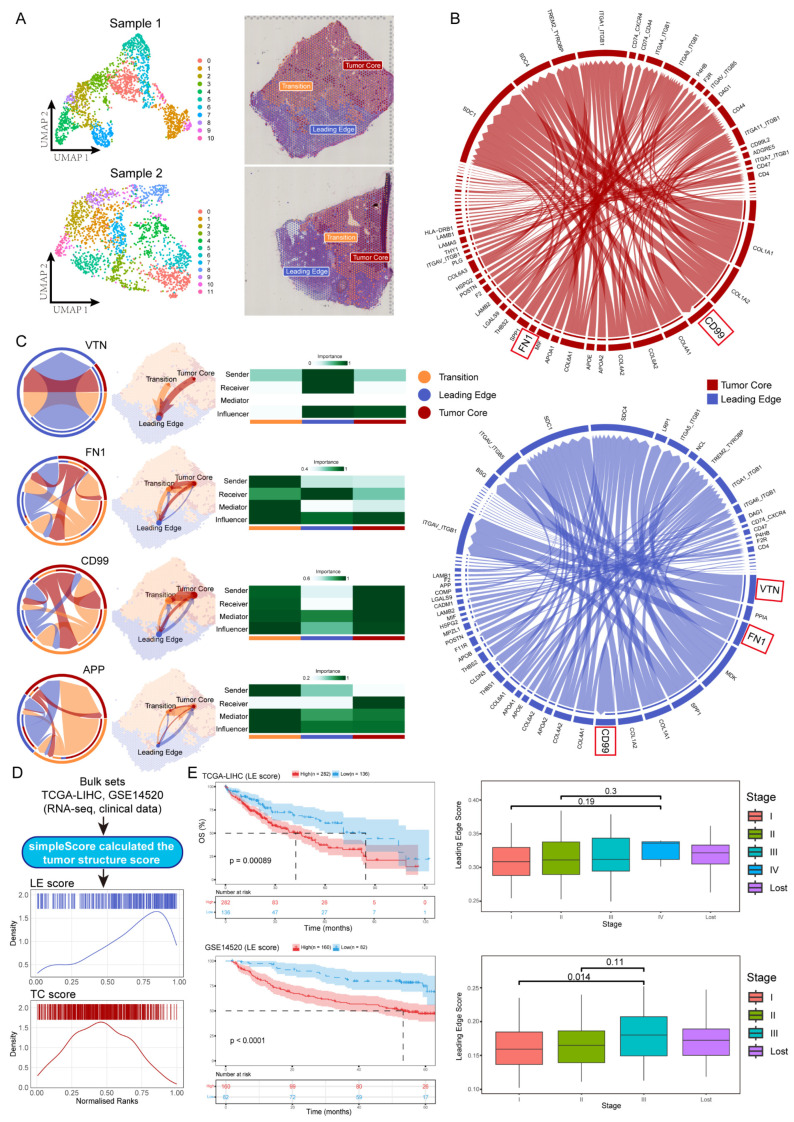
Identify the spatial structure of the tumor and reveal the biological functions of different regions based on pathological indicators. (**A**) Distribution of spatial spot information in patients and distribution of spatial structural regions on tissue sections. (**B**) String diagram showing the level of molecular interactions between TC and LE regions. (**C**) Expression intensity of molecular signaling axes between different regions. (**D**) Schematic diagram of calculating patient LE and TC scores. (**E**) Survival analysis of the LIHC cohort and GSE14520 data based on LE scores, and plotting of tumor staging box plots.

Conventional single-cell technologies are inadequate to resolve dynamic evolution patterns of spatially structured regionalization-derived intratumor heterogeneity. In this study, ST technology is integrated with quantitative histopathological analysis to systematically unveil the entire spatial structural features of HCC. On tissue sections of 2 HCCs, we detected 1733 tumor LE regions, 1888 transition regions, and 627 TC regions. Their spatial distribution profiles were mapped with spatial mapping methods (Figure 3A). The complex interactions among tumor regions show that TC and LE regions take the center position in cellular signaling. TC controls tumor biology by governing cell death and immune function in local TIME by FN1 signaling and CD99 signaling pathways. In contrast, LE markedly increases VTN and FN1 signaling activity, which stimulates migration and proliferation but decreases cell adhesion, thereby modulating stromal cell remodeling to induce angiogenesis, enhancing LE region-invasive ability (Figure 3B and Figure S4). Being deeply involved in tumor invasiveness and immune regulation of the LE region, we also performed signal flow analysis. VTN, FN1, and CD99 signaling were found to be actively enriched in this region, and these activated signals contributed to a TIME where cell adhesion was lost, and cell migration and extracellular matrix remodeling were enabled, eventually leading to an invasive phenotype (Figure 3C) [16]. Significant enrichments of CAFs, extracellular matrix, and pro-tumor features were detected in the LE region. The TC region also exhibited ECM- and pro-tumor-associated transcriptional activity. However, compared with LE, TC showed a distinct TIME composition and did not exhibit the same relative enrichment of the combined TIME-ID and TIME-IS programs. Therefore, the stromal and immune-suppressive features in TC may differ in extent and cellular context from those observed in LE [17]. In contrast, high-level expression of proangiogenic, co-stimulatory molecules and tumor-promoting factors within TC helps to maintain tumor cell survival and proliferation by activation of angiogenic pathways for hypoxic microenvironment adaptation. Functional analysis suggests that the TC region creates an inhibitory immune microenvironment that favorably affects Th1/Th2 cell differentiation and regulates the chemokine network. It aggravates the fibrotic process and facilitates immune evasion by dysregulating the MAPK and p53 signaling pathways. Anomalous enhancement of CGMP/PKG molecular pathways and the cell-vascular interaction network facilitates angiogenesis and proliferation in TC to LE, which can dramatically enhance cancer cell interstitial permeability by cytoskeletal remodeling and profound Rap1 signaling activation to promote tumor invasive capability. Simultaneously, the disorder-activating PI3K/AKT signal pathway represses tumor cell apoptosis, sustains tumor energy metabolism, and works synergistically in tumor proliferation and metastasis. Excessive EMT and cell cycle processes can disrupt the restrictive intercellular connections, allowing cancer cells to increase tissue permeability. The disruption of intercellular barriers enables cancer cells to invade and escape from the original microenvironment (Figure S5A,C). To evaluate the clinical impact of pathologically annotated LE and TC regions, we carried out a patient-based survival analysis based on LE and TC scores (Figure 3D and Figure S5B,D). Results indicated that patients with higher LE scores demonstrated overall worse prognosis, and the LE scores positively correlated with the disease staging, especially for tumor metastasis in the GSE14520 cohort; patients with high LE scores showed a very significantly higher risk of metastasis (Figure 3E).

### 2.4. Constructing a Spatiotemporal Map of Hepatocellular Carcinoma

**Figure 4:**
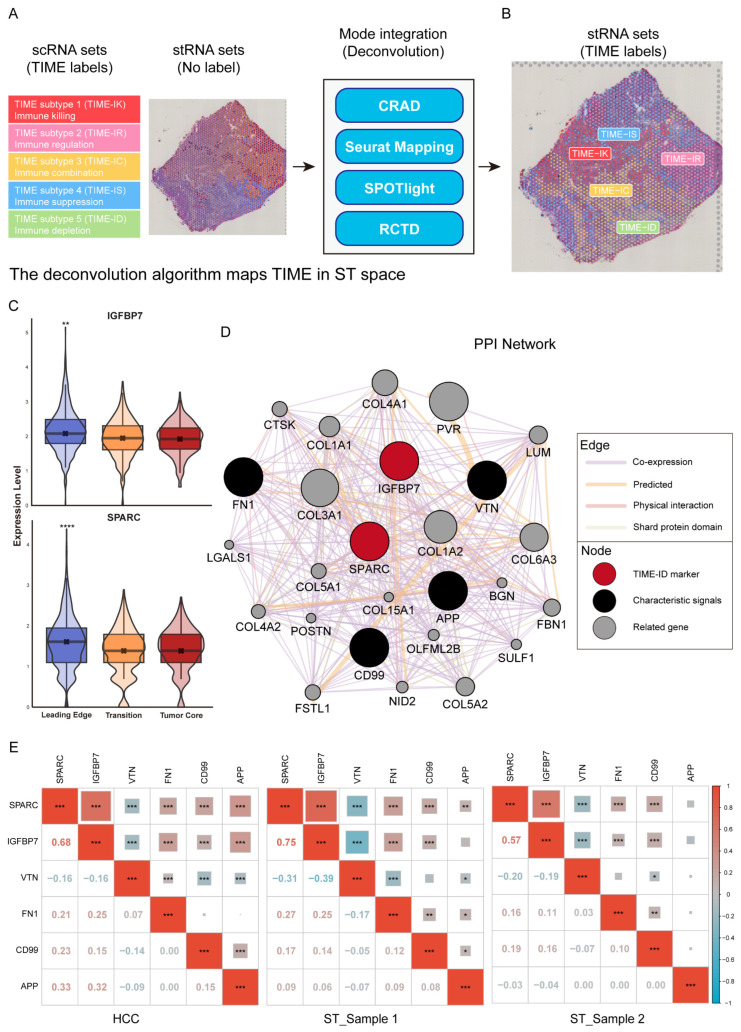
Deconvolution mapping and molecular interaction network analysis of TIME subtypes. (**A**) Mapping of the TIME subtype using four deconvolution algorithms. (**B**) Distribution of the TIME subtype in tissue sections from different samples. (**C**) Box plot of the expression of TIME-ID subtype marker genes in spatial structures. (**D**) Protein interaction network between TIME-ID subtype marker genes and key pathway molecules. (**E**) Correlation heatmap showing the level of molecular co-expression correlation at single-cell expression spectrum and spatial expression spectrum resolution.

Due to the fact that the molecular expression patterns extracted from the TIME signaling pathway analysis are highly overlapping with the signaling patterns between spatial structure, we hypothesize that the biological characteristics of the tumor structure are closely related to the specific composition pattern of TIME. To test this hypothesis, we first integrate four deconvolution algorithms and map the immune subtypes of tumor precisely to spatial tissue structure (Figure 4A). According to the proportional abundance of each subtype within each different type of Spot cluster, we characterized their spatial localization and composition profiles (Figure 4B and Figure S6A). The results show that tumor LE enriched TIME-ID and TIME-IS subtypes exclusively, involved in tumor stromal-cell signaling by the APP pathway and CD99 pathway for interaction between cancer cells and stromal cells. This reduces the adhesiveness of cancer cells and stimulates migratory activity, culminating in malignant invasion. At the same time, cancer cells co-regulate their adjacent clusters by secreting VTN and FN1 cues that create a depleted microenvironment that further facilitates tumor immune evasion and invasiveness. Moreover, the spatial enrichment scores of TIME-ID marker genes (IGFBP7, SPARC) reveal their strong expression in the LE region, which further verifies the spatial localization results (Figure 4C). SPARC contributes to extracellular matrix formation and facilitates cancer cell shape changes. Regulates the process of communication with extracellular matrix cells through the elimination of focal adhesion, thus aiding in generating an anti-adhesive effect on cells, leading ultimately to the invasive ability of tumor cells [18]. IGFBP7 supports tumor cell proliferation, induces angiogenesis and EMT pathways, and the high expression of that protein inhibits infiltrations of lymphocytes and eventually worsens the patient’s prognosis [19]. Protein–protein interaction and co-expression analyses indicated that SPARC and IGFBP7 were connected to genes involved in the inferred LE-associated signaling network (Figure 4D). Expression of SPARC and IGFBP7 was negatively correlated with VTN expression, indicating that cancer cells might regulate signaling of this pathway to collaboratively establish the depleted microenvironment by controlling related functions of TIME-ID subtype molecular functions. Also, there was a strong positive relationship between both genes and other signaling molecules, suggesting that TIME-ID subtype enhances FN1 pathway activity synergistically through activating APP and CD99 signaling to further contribute to tumor invasive phenotypes. Consistent co-expression patterns across multi-omics samples further validate that SPARC and IGFBP7 emerged as candidate LE-associated genes whose expression patterns were correlated with TIME-ID enrichment and inferred intercellular communication networks. These observations suggest a potential role for these genes in LE-associated stromal remodeling and immune depletion, which warrants direct functional validation (Figure 4E).

### 2.5. SpaPred Predicts Hepatocellular Carcinoma Immune Microenvironment and Spatial Structure Distribution

**Figure 5:**
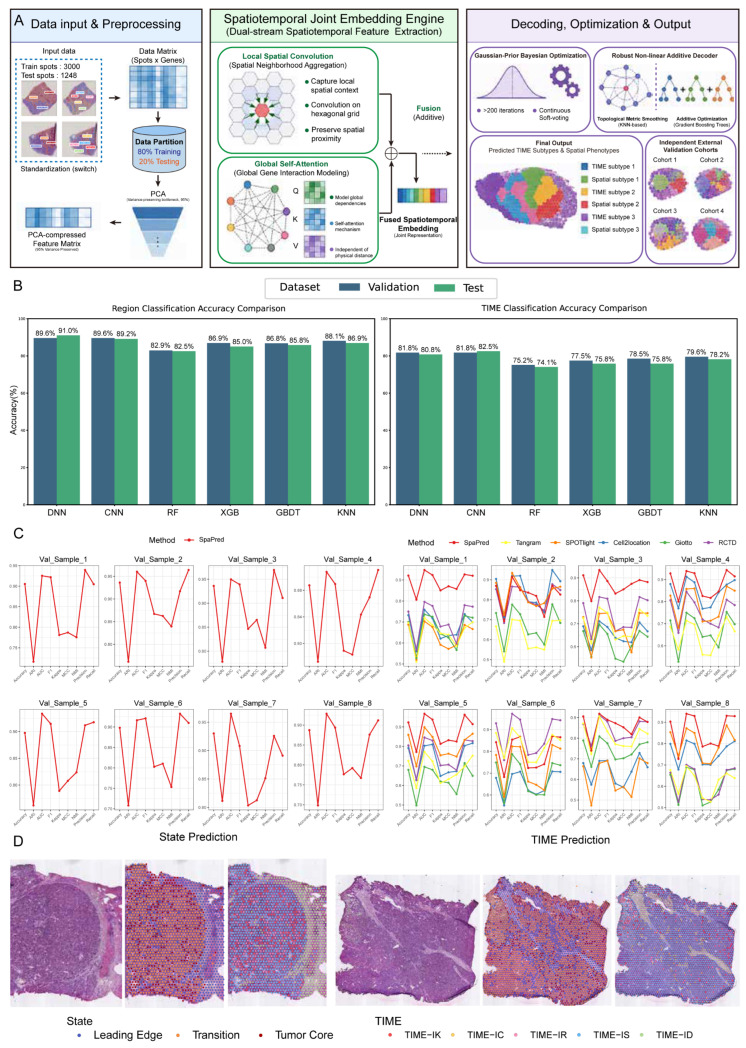
Performance and prediction results of the SpaPred model. (**A**) Schematic diagram of the SpaPred model construction. (**B**) Prediction accuracy of a single model on the validation set and test set. (**C**) SpaPred was compared with Cell2location, RCTD, SPOTlight, Tangram, and Giotto across the analyzed spatial transcriptomic datasets. For methods that output cell-type or subtype abundance, the dominant TIME subtype was assigned according to the maximum inferred abundance. For methods that output mapped cells or spatial clusters, TIME and spatial-region labels were assigned using predefined subtype marker signatures and histology-informed spatial annotations. All methods were evaluated on the same spatial spots within each dataset. (**D**) Distribution pattern of model predictions for external source samples in the organization.

The profound intrinsic heterogeneity of tumors, coupled with the intricate diversity of spatial architectures and the highly complex TIME, poses formidable challenges for accurately decoding ST data in HCC. To overcome these computational bottlenecks, we engineered SpaPred, an integrated spatiotemporal representation learning framework (Figure 5A). In the test set, the average prediction accuracy of the single model for spatial structure and TIME subtype reached 86.7% and 77.9%, respectively (Figure 5B). The SpaPred model, which uses Bayesian dynamic weight allocation fusion, achieved outstanding performance in terms of prediction accuracy and generalization ability, with an AUC value of no less than 0.95 and an AP value of no less than 0.8. Additionally, SpaPred outperformed the single model in terms of prediction accuracy and F1 weight, clearly demonstrating that the fusion model can fully leverage the advantages of the single model to achieve more efficient performance. In spot-level ten-fold cross-validation, SpaPred achieved mean accuracies of 91.3% and 82.7% for spatial-region and TIME-subtype classification, respectively, with corresponding weighted F1 scores of 0.912 and 0.826 (Figure S6B). To further validate the robustness and superiority of SpaPred, we conducted a comprehensive benchmark analysis of various state-of-the-art methods across eight independent spatial transcriptomics datasets (Figure 5C). As shown in the figure, SpaPred consistently demonstrates high spatial structure annotation performance across all datasets. In the available validation datasets, SpaPred showed relatively consistent performance, suggesting that the framework may have potential for handling dataset-specific heterogeneity. However, validation in larger and more diverse spatial cohorts is required. Regarding predictive performance for TIME subtype classification, SpaPred also demonstrates a significant advantage over competing methods. Across evaluation metrics such as accuracy, F1 score, MCC, ARI, NMI, Kappa, and AUC, SpaPred outperforms both traditional spatial deconvolution methods and machine learning-based approaches. Notably, SpaPred achieves a better balance between precision and recall, as evidenced by its consistently high F1 scores, indicating that the model possesses strong robustness when handling class imbalance and complex immune components in the tumor microenvironment. Furthermore, the performance of SpaPred improvements is not limited to individual metrics but is validated across multiple complementary evaluation criteria. This multidimensional advantage demonstrates that SpaPred not only enhances predictive accuracy but also improves the stability and interpretability of spatial pattern recognition. These benchmark results support the potential utility of SpaPred for decoding spatial organization and immune-associated heterogeneity in the analyzed HCC datasets. Its robustness across broader HCC cohorts remains to be established. To demonstrate the clinical applicability of the SpaPred model, the built-in Predict function was used to predict HCC samples from two external sources, and the results showed that the model can clearly predict the spatial structure of the tumor and the distribution patterns of corresponding TIME subtypes (Figure 5D).

### 2.6. Prediction of Candidate Drugs Targeting the Leading Edge of Hepatocellular Carcinoma

**Figure 6:**
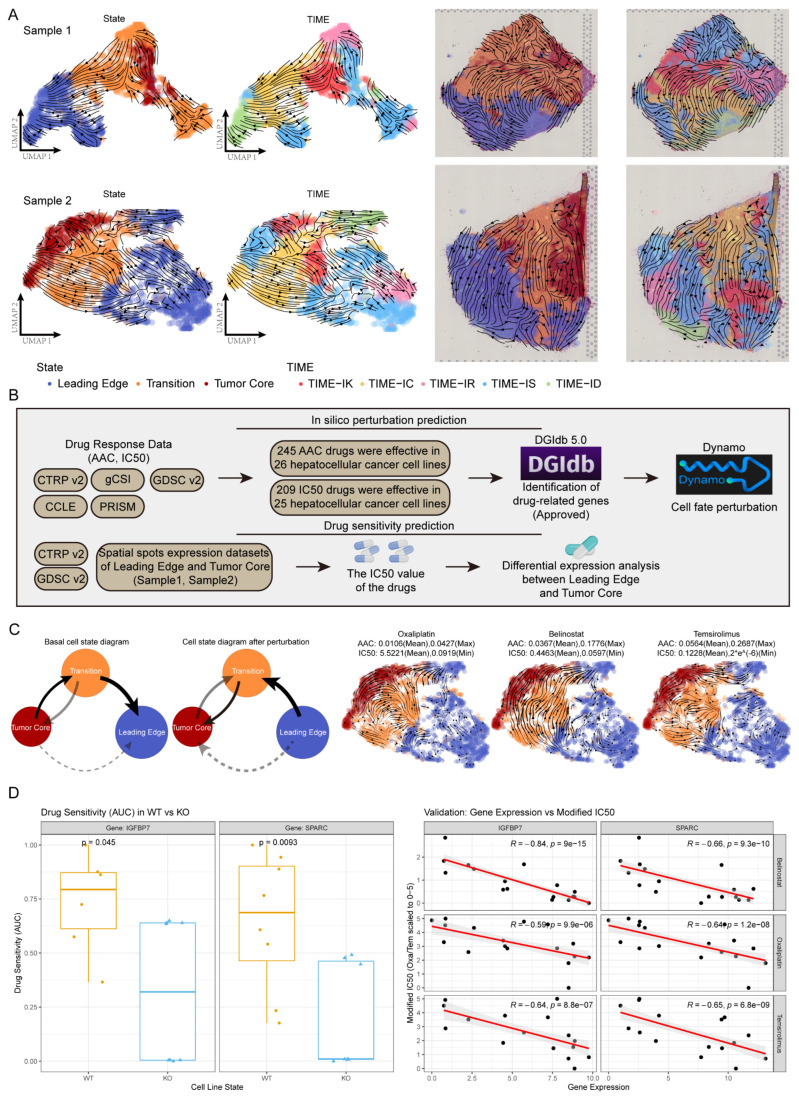
Tumor developmental trajectory analysis and screening of drug candidates targeting the LE region. (**A**) RNA-velocity-based visualization of inferred spot-level transcriptional-state relationships among TC-, transition-, and LE-associated spots and among TIME subtypes in low-dimensional and spatial embeddings. (**B**) Schematic diagram of drug candidate prediction combining in silico perturbation algorithms and drug sensitivity analysis models. (**C**) Schematic diagrams of cellular developmental patterns before and after algorithmic perturbation, and developmental trajectories of candidate drugs identified through trajectory inversion screening. (**D**) Box plots show IC50 values in HCC cell lines before and after perturbation of SPARC or IGFBP7. Scatter plots show correlations between SPARC/IGFBP7 expression and IC50 values for the prioritized compounds. Lower IC50 values generally indicate greater drug sensitivity.

Based on a transcription kinetic analysis using the dynamic model and RNA velocity, this study reveals the overall spatiotemporal evolutionary trajectory of HCC: RNA-velocity and vector-field analyses suggested a computational directional relationship among spot-level transcriptional states associated with TC, transition, and LE regions. Cells in the core and transitional region exhibit high plasticity, activating transcription of genes related to proliferation and signaling to respond to external stimuli, demonstrating significant potential for phenotypic conversion. In the LE region, cells have largely completed core gene transcription and entered the protein translation phase, with enhanced intercellular interactions forming the microenvironmental foundation supporting invasion and metastasis. Through the evolutionary process of TIME, the primary initial state has the immune-competent subtypes mainly distributed in the core and transitional region, and cells communicate closely with cancer cells by differentiating immune-related features. The end state has the main distribution of TIME-ID and TIME-IS subtypes, which regulate gene products to create the lost microenvironment inside the LE region. This further propels tumor invasiveness and metastasis, guiding the malignant development of intratumoral heterogeneity (Figure 6A). We may look forward to targeted therapy against tumor LE as one of the most effective therapeutic strategies to suppress metastasis and reduce recurrence, in addition to improving the efficiency of the treatment for HCC. By the Dynamo in silico perturbation dynamics model, integrating with sensitivity analysis algorithms, to systematically screen candidate therapeutic drugs against the LE. Among HCC cell lines with available pharmacogenomic data, 245 compounds had eligible AAC measurements and 209 compounds had eligible IC50 measurements after exclusion based on the missing/zero drug-response measurement criterion. AAC- and IC50-based analyses were performed separately, and 76 compounds were supported by both metrics (Figure S7). We input the validated drug targets into the Dynamo algorithm and predicted the general trend of cellular developmental trajectory after drug perturbation by simulating the variation of the cellular state vector field. Predicted IC50 values differed between LE-associated and non-LE-associated spots for selected compounds, suggesting potential regional variation in drug-response profiles (Figure 6B). Compounds predicted to perturb the inferred TC–transition–LE-associated transcriptional-state relationship and to show favorable LE-associated pharmacogenomic profiles were retained as candidate compounds for further evaluation. After strict screening, we finally obtained 9 drugs that could strongly affect the evolutionary trajectory and exhibited strong sensitivity. To evaluate their clinical prospect, we combined several databases of drug information. From HCC treatment scores, number of side effects, and clinical availability score, Oxaliplatin (the approved drug) was recognized as an active drug for HCC clinical treatments. Although Oxaliplatin, Belinostat, and Temsirolimus showed predicted perturbation effects on LE-associated trajectories in the computational analysis, their known pharmacological targets are distinct. Finally, Oxaliplatin, Belinostat, and Temsirolimus were prioritized as candidate compounds for further experimental evaluation in LE-associated HCC models (Figure 6C). To further validate the reliability of the predicted drugs in targeting the LE molecular mechanism of tumors, we conducted a functional association analysis of the LE-specific key genes SPARC and IGFBP7 in HCC cell lines based on drug sensitivity data collected before and after large-scale gene knockout. The results showed that after SPARC and IGFBP7 expression was suppressed, the sensitivity of most HCC cell lines to the candidate drugs was significantly enhanced, as evidenced by a significant decrease in IC50 values, suggesting a potential association between SPARC/IGFBP7 perturbation and the drug-response phenotype in the analyzed cell-line datasets. Further correlation analysis indicated that the IC50 values of Oxaliplatin, Belinostat, and Temsirolimus were significantly negatively correlated with the expression levels of SPARC and IGFBP7; that is, as the expression levels of these two LE marker genes increased, cellular sensitivity to the aforementioned drugs significantly increased. These pharmacogenomic analyses suggest that SPARC/IGFBP7-associated transcriptional states may be related to drug-response heterogeneity in HCC cell lines. However, the direction of the associations varied across analytical contexts, and the current data do not establish whether SPARC or IGFBP7 promotes sensitivity or resistance. Their mechanistic contribution to drug response remains undetermined (Figure 6D). In summary, these results indicate that the three prioritized candidate drugs not only demonstrate significant effects in kinetics-based interventions but also act on LE-specific molecular features. These compounds may represent candidates for investigating whether pharmacological perturbation of LE-associated transcriptional programs can modulate HCC progression. However, their effects on the LE microenvironment, their relationship with SPARC/IGFBP7 activity, and their therapeutic relevance require validation in experimental models.

## 3. Discussion

Intratumoral heterogeneity is closely related to poor prognosis, which may be caused by the diversity of the tumor cellular composition and the coexistence of various complex intercellular relationships that integrate the comprehensive biological behavior of the tumor. While knowledge of HCC intratumoral heterogeneity has improved in the past few years, the relevant mechanisms of spatiotemporal tumor heterogeneity and the biological rules that control dynamic evolution remain to be further explained [20,21,22,23,24].

To overcome these limitations, this study systematically mapped the spatiotemporal heterogeneity of hepatocellular carcinoma by integrating multi-omics data, revealing the pivotal role of the tumor leading edge as a key spatial structure driving malignant progression. Our findings indicate that the tumor leading edge is not passively occupied by invasive cancer cells, but rather actively reshapes the local microenvironment with elevated expression of genes such as SPARC and IGFBP7, which was associated with inferred VTN, FN1, APP, and CD99 related signaling networks and stromal remodeling programs, thereby reducing cancer cell adhesion, enhancing migration capacity, and simultaneously establishing a matrix barrier formation mechanism characterized primarily by the TIME-ID subtype [25,26]. This mechanism tightly integrates the intrinsic invasiveness of tumor cells with the extrinsic immune-depleted (suppressive) microenvironment, providing a new spatiotemporal perspective for understanding why HCC exhibits greater malignant potential in specific regions (LE). Notably, previous studies have extensively reported the roles of SPARC and IGFBP7 in stromal remodeling across various tumors [27,28]. However, these studies are largely based on single-omics data, such as bulk or single-cell sequencing, making it difficult to capture their spatial colocalization and functional synergies in situ within tissues, as well as the role of the immune microenvironment in this process. The core contribution of this study lies in demonstrating, through spatiotemporal transcriptomics in HCC tissues, that these gene expression events exhibit a highly specific spatial overlap with the TIME-ID microenvironment within the tumor LE region, rather than being randomly distributed throughout the tumor. The regional association of these transcriptional programs suggests that the LE may serve as a spatially organized microenvironmental niche linking stromal remodeling, immune depletion, and invasive tumor features, connecting multi-cellular signaling regulatory networks and integrating malignant features such as stromal remodeling and immune evasion within the same microenvironment, thereby synergistically driving tumor progression. This discovery elevates the understanding of individual molecular functions to a system-level perspective characterized by spatiotemporal organization. From a methodological perspective, existing spatial deconvolution tools such as Cell2location, RCTD, and Giotto primarily rely on single-cell reference matrices to infer cell type composition; these are essentially estimates of cell proportions and cannot directly identify spatial domains with specific biological functions. In contrast, the SpaPred model is capable of performing comprehensive feature identification on ST data while simultaneously outputting information on the distribution of tissue types and TIME subtypes. Benchmark results demonstrate that SpaPred significantly outperforms existing methods in terms of accuracy and robustness when identifying tumor LE regions with complex boundaries, and exhibits excellent generalization capabilities across datasets from different sequencing platforms. This technical advantage makes it suitable not only for expression profiling of HCC samples but also holds potential for rapid spatial phenotyping of prospective clinical samples, providing a viable tool for real-time analysis of spatial heterogeneity in the tumor microenvironment. At the translational medicine level, this study integrated pharmacogenomics databases to screen three candidate drugs (Oxaliplatin, Belinostat, and Temsirolimus) that can target the evolutionary trajectory of the leading edge [29]. Among these, Oxaliplatin has been evaluated and used in systemic chemotherapy regimens for selected patients with advanced HCC; meanwhile, Belinostat and Temsirolimus exhibited similar potential for trajectory intervention as Oxaliplatin in this study. The three prioritized compounds are pharmacologically distinct and may potentially influence different components of the LE-associated ecosystem. Oxaliplatin is a platinum-based cytotoxic agent that induces DNA damage and may preferentially affect proliferative malignant-cell populations; in some contexts, it can also promote immunogenic cell death [30]. Belinostat is a histone deacetylase inhibitor and may influence transcriptional plasticity, epithelial–mesenchymal transition, extracellular-matrix remodeling, and immune-regulatory gene expression through epigenetic modulation [31]. Temsirolimus inhibits mTORC1 and may interfere with growth-factor-responsive metabolic, survival, and PI3K/AKT/mTOR-related programs that are associated with malignant-cell adaptation and stromal activation [32]. The clinical translation of these candidates should be interpreted cautiously. Oxaliplatin, Belinostat, and Temsirolimus are systemically administered, mainly intravenous agents, and none has been validated as LE-specific. Oxaliplatin may cause neuropathy and myelosuppression, whereas Belinostat and Temsirolimus may cause hematologic, gastrointestinal, metabolic, or infectious adverse effects. Moreover, resistance through DNA repair, epigenetic adaptation, or PI3K/AKT/mTOR/MAPK bypass signaling may limit efficacy. Thus, these drugs should be considered computationally prioritized candidates requiring preclinical validation of safety, resistance, and effects on LE-associated programs. This finding suggests that existing drugs may exert their targeted therapeutic effects by reshaping the spatial structure of the LE region, rather than solely through direct cytotoxicity. Future studies could utilize organoid or PDX models to verify whether these drugs can reverse the immune exhaustion phenotype in the LE region or inhibit stromal remodeling, thereby providing experimental evidence for their application in precision therapy for HCC. In summary, this study employed a spatiotemporal multi-omics integration strategy to reveal the molecular mechanisms by which the LE region of HCC tumors, as a spatial functional unit, drives malignant progression, and developed the SpaPred model to enable precise analysis of tumor spatiotemporal heterogeneity. This research framework offers new insights into the spatial organization of solid tumors and lays a theoretical foundation for the development of targeted therapeutic strategies based on spatial structure.

While our study provides a computational framework (SpaPred) for deciphering the spatiotemporal architecture of the TIME in HCC, several limitations should be acknowledged. An important limitation of this study is the relatively small number of spatial transcriptomic samples. In particular, the training cohort included only two HCC specimens, while the external validation cohort consisted of eight specimens. Although the independent validation samples supported the reproducibility of several major spatial patterns, this sample size is insufficient to capture the full inter-patient diversity of HCC spatial architecture, especially the complex and irregular boundaries of tumor leading-edge regions. In addition, spot-level spatial transcriptomic data are subject to spatial autocorrelation, which may lead to optimistic performance estimates in internal validation. Therefore, the current performance of SpaPred should be interpreted as preliminary. Future studies should validate the framework in larger, multi-center cohorts with standardized pathological LE annotations and higher-resolution spatial transcriptomic or spatial proteomic data. Future studies should evaluate continuous LE and TIME scores in adequately powered univariable and multivariable Cox proportional-hazards models, test the proportional-hazards assumption, account for multiple comparisons, and validate pre-specified cut-offs in independent clinically annotated cohorts. Although we utilized extensive independent multi-omics cohorts and large-scale pharmacogenomic databases (e.g., GDSC, CTRP, gCSI, PRISM, and CCLE) for orthogonal validation, the therapeutic efficacy of our prioritized drug candidates (Oxaliplatin, Belinostat, and Temsirolimus) targeting the LE spatial niche remains to be definitively confirmed using patient-derived organoids (PDOs), patient-derived xenograft (PDX) models, or specific cell viability assays. Furthermore, the structural and functional cellular communications proposed in this study were inferred using computational correlation networks. Establishing definitive causality and direct physical binding will require future functional perturbation models and physical interaction studies, such as co-immunoprecipitation (Co-IP). Finally, the exact localized protein expression and physical cellular interactions within the LE microenvironment ultimately require validation in prospective, internal clinical cohorts utilizing high-resolution, protein-level spatial techniques, such as multiplex immunofluorescence (mIF) or spatial mass cytometry. Therefore, the SpaPred framework and its downstream findings should be viewed as a robust, hypothesis-generating tool that lays the necessary groundwork for future targeted experimental and translational clinical investigations.

## 4. Materials and Methods

### 4.1. Data Collection and Preprocessing

In this study, we collected 12 single-cell transcriptomic datasets (141 primary HCC samples) from the Gene Expression Omnibus (GEO) [33] and the China National Center for Biological Information (CNCB), and 2 external validation datasets (38 primary HCC samples). After integration and quality control (nCount_RNA > 1000 & nFeature_RNA > 500 & log10GenesPerUMI > 0.8 & percent.mt < 8) of the training dataset using the R package Seurat (v5.1.0) [34] for downstream analysis (Figure S1A). Highly variable genes were identified using the SCTransform method, and the appropriate number of principal components was selected based on the elbow plot, after which batch effects were corrected using Harmony (Figure S1B) [35]. Following the construction of a nearest neighbor graph, dimensionality reduction was performed using UMAP, and clustering analysis was conducted with the Louvain algorithm. Putative malignant epithelial cells were identified using inferCNV (v1.18.1) [36], with endothelial cells used as the non-malignant reference population. CNV scores were calculated for epithelial-cell clusters based on their inferred transcriptional deviation from the endothelial-cell reference profile; higher CNV scores indicated a greater inferred CNV burden. The mean − 2 SD threshold was used to identify epithelial clusters with exceptionally low CNV scores among epithelial clusters. Clusters below this lower-bound threshold were classified as putative non-malignant epithelial clusters, whereas the remaining epithelial clusters with relatively higher CNV scores were classified as putative malignant epithelial clusters. This classification was interpreted together with canonical epithelial and hepatocyte marker expression. Additionally, a primary HCC dataset comprising 242 patients was obtained from GEO, and a LIHC-cohort dataset from The Cancer Genome Atlas (TCGA) database, which includes 418 patients. We removed samples with missing clinical follow-up information and retained only patient data with complete prognostic information. The spatial transcriptomics (ST) datasets included a training set of 2 samples and an external validation set of 8 samples. SpaceRanger (v3.1.1) was used to map raw FASTQ files to the GRCh38 reference genome. Based on Velocyto (v0.17.16) [37], the BAM files were converted into Loom files for downstream analysis. Following Seurat preprocessing, histological annotations of tissue sections were combined with spatial clustering analysis. This approach identified histological structural composition based on pathological discriminative indicators (including cell density, nuclear morphology, H&E staining intensity, nuclear-to-cytoplasmic ratio, and mitotic activity. Spatial regions were annotated using H&E images in combination with tissue morphology and spatial location. The tumor core (TC) was defined as the central tumor area located away from the tumor–non-tumor interface and characterized by a compact tumor-cell architecture. The transition region was defined as the intermediate zone between TC and the tumor boundary. The leading edge (LE) was defined as the peripheral tumor area adjacent to the tumor–non-tumor interface, with an irregular or infiltrative boundary and increased stromal association, alongside histological features recognized by the QuPath semi-automated characterization software. Detailed information on all datasets and their analytical roles is provided in Table S2.

### 4.2. Identification of Tumor Immune Microenvironment Subtypes

To characterize the spatiotemporal integration of cells in HCC, we employed the corr. test function and Euclidean distance to assess correlations between cell subtypes. Based on the Ward D clustering algorithm, we identified five distinct cellular modules and completed subtype characterization using four criteria: (1) biological function of the cell subtype, (2) integration of module functions, (3) association with clinical prognosis, (4) expression levels of literature-derived immune biomarkers (Table S1). We quantified the expression levels of TIME markers across modules using the AddModuleScore function and visualized the expression patterns of differentially expressed genes (DEGs) among different subtypes by radar plots. Further Gene Ontology (GO) [38] and Kyoto Encyclopedia of Genes and Genomes (KEGG) [39] pathway enrichment analyses were performed on the DEGs. We preprocessed the external dataset based on the same quality control standards, determined the subtype composition of the 50 top DEGs, and removed the background noise calculated by 100 randomly selected genes. The performance of TIME classification was comprehensively evaluated based on the independent expression levels of subtype marker genes, pathway enrichment results, and prognostic associations.

### 4.3. Correlation Analysis Between TIME Subtypes and Clinical Characteristics

Survival associations of TIME, LE, and TC scores were explored using Kaplan–Meier analysis and log-rank tests. Score thresholds were determined using the surv_cutpoint approach implemented in the Survival-related R package. Because these thresholds were derived from the analyzed cohorts, the survival analyses should be interpreted as exploratory and do not establish clinically validated cut-offs or independent prognostic effects. Additionally, the expression heatmaps visualized correlations between TIME subtypes and other clinical variables (age, sex, clinical stage).

### 4.4. TIME Deconvolution at Spatial Resolution

To explore the heterogeneity of TIME subtypes across different tumor spatial organizations, we integrated four deconvolution algorithms to map TIME subtypes described by scRNA transcriptomic data onto ST objects: (1)CARD (v1.1) [40] constructed a reference matrix using a conditional autoregressive model and incorporated spatial similarity via non-negative matrix factorization (NMF) [41]. (2) FindTransferAnchors identified cross-modal anchors between scRNA and ST references. (3) SPOTlight (v1.6.7) [42] performed seeded NMF regression to map subtypes to spatial structures. (4) Robust Cell Type Decomposition (RCTD) algorithm in the spacexr (v2.2.1) [43] integrated scRNA and ST data to infer cell type proportions. For the probabilities output by the algorithm, we characterize each Spot’s dominant TIME subtype based on the mode and evaluate the accuracy and generalization of the deconvolution by comparing and analyzing pathway enrichment results across subtypes.

### 4.5. The SpaPred Model

To systematically assess the spatiotemporal heterogeneity of HCC, we developed SpaPred, a deep learning framework specifically designed for ST datasets. First, to filter out inherent transcriptomic noise while preserving key biological signals, the raw expression matrix is projected onto a low-dimensional orthogonal subspace (Z_base_), a process that retains 95% of the variance information. The core architecture of SpaPred is driven by spatiotemporal joint embedding. For each spatial spot i, its local spatial dependencies are aggregated via a spatial convolution operator, defined as follows:

$$Z_{local\left(i\right)}=\;\sigma \;\sum \left[W_{s}\times Z_{base}\left(j\right)\right]+b_{s}$$

where j belongs to the neighborhood N(i), W_s_ represents spatial filters, and σ is the activation function.

Concurrently, to decipher the global gene-gene interaction networks devoid of spatial distance constraints, we integrated a non-local self-attention mechanism. The global context vector is computed as:

$$Z_{global}=Softmax\left(\frac{Q\times K^{T}}{\sqrt{d_{k}}}\right)\times V$$

where Q, K, V are derived from linear transformations of Z_base_.

The local and global representations were first projected to the same latent dimension d. They were then integrated by element-wise addition to generate the fused representation: Z_fused_ = Z_local_ + Z_global_. where Z_local_, Z_global_, Z_fuse_ ∈ R^N∗d^, N is the number of spatial spots, and d is the latent feature dimension. The previous description of this operation as concatenation was corrected. To map this complex manifold to distinct TIME subtypes and spatial phenotypes, we designed a Robust Non-linear Additive Decoder. To inherently resist spatial dropouts and extreme class imbalances prevalent in ST data, this decoder formulates the prediction Y_pred_ through an additive gradient optimization protocol intertwined with a localized topological metric smoothing function:

$$Y_{pred}=\;\sum \left[\alpha_{m}\times \phi_{m}\left(Z_{fused}\right)\right]+\beta \times M_{topo}(Z_{fused})$$

where ϕ_m_ represents sequential non-linear recursive partitioning functions, and M_topo_ enforces label consistency based on latent spatial distances.

The entire hypothesis space is dynamically governed by a Gaussian-Prior Bayesian Optimization Module. The framework models the objective function as a Gaussian Process GP(μ, k). Through more than 200 acquisition-driven probabilistic iterations, the optimal feature weights are seamlessly calibrated and fused via a continuous soft-voting strategy. Ultimately, the generalization capacity of SpaPred was validated across 8 independent external ST samples. Supported by a unified data-preprocessing pipeline, the spatial architectures predicted by our mathematical model were histopathologically validated and visualized using the SpatialPlot function in the Seurat suite.

To systematically predict histology-informed spatial-region labels and TIME subtypes from spatial transcriptomic data, we developed SpaPred, a multi-model ensemble classification framework. SpaPred jointly generated predictions for two classification tasks: spatial-region classification, including TC, transition region, and LE, and TIME-subtype classification.

The input data were organized as spot-by-gene expression matrices. Because the input expression matrices had been pre-scaled, additional standardization was not applied. Principal component analysis (PCA; scikit-learn) was performed using the training data, and the number of principal components was fixed at 50. Thus, each spot was represented by a 50-dimensional transcriptomic feature vector. The PCA transformation fitted on the training set was subsequently applied to the validation and test sets.

The training dataset was randomly divided into an 80% training subset and a 20% validation subset using train_test_split, with stratification according to the spatial-region labels and a fixed random seed of 110. The independent test set was not used for model fitting or ensemble-weight optimization. For ten-fold cross-validation, the complete dataset was partitioned using StratifiedKFold with 10 folds, shuffling, and a random seed of 110.

SpaPred included six base classifiers: a one-dimensional convolutional neural network (CNN), an attention-based deep neural network (DNN), random forest (RF), XGBoost (XGB), gradient boosting decision tree (GBDT), and k-nearest-neighbor (KNN) classifiers. The attention-based DNN received a 50-dimensional PCA feature vector as input. The first shared layer consisted of a fully connected layer mapping the input features to 1024 hidden units, followed by ReLU activation, batch normalization, and dropout (dropout rate = 0.45). An eight-head attention layer was then applied to this latent representation, followed by a residual connection. The second shared layer mapped the features from 1024 to 512 units and included ReLU activation, batch normalization, and dropout. Each task-specific branch consisted of a 256-unit fully connected layer with ReLU activation, batch normalization, and dropout, followed by an output layer with either three spatial-region classes or five TIME-subtype classes.

The CNN treated the 50-dimensional PCA feature vector as a one-dimensional input sequence. The network consisted of two convolutional blocks with 128 and 256 channels, respectively. Each block used a kernel size of 3, ReLU activation, batch normalization, max pooling, and dropout (dropout rate = 0.30). The flattened output was processed through a shared fully connected layer with 512 units, ReLU activation, batch normalization, and dropout. Two task-specific branches, each containing a 256-unit hidden layer followed by a classification layer, generated three spatial-region logits and five TIME-subtype logits, respectively.

### 4.6. The Benchmark Test

To systematically evaluate the performance differences between SpaPred and existing spatial transcriptomics deconvolution methods, this study conducted benchmarking analyses using both internal and external datasets. The data included a single-cell transcriptomics reference dataset and 8 independent spatial transcriptomics validation samples. All data underwent a standardized preprocessing workflow, including normalization, clustering analysis, and identification of TIME subtypes and spatial structures, to ensure comparability of results across different methods. The single-cell dataset was used to construct a reference feature matrix or to train models for five representative deconvolution methods (Cell2location [44], RCTD, SPOTlight, Tangram [45], and Giotto [46]), with all implementations adhering to the official parameter settings recommended by each method. For SpaPred, a pre-trained ensemble model was used to perform direct inference on each validation dataset to obtain prediction results. For deconvolution-based methods, cell type abundance matrices were inferred independently on each validation dataset, and the dominant TIME subtype at each spatial location was determined based on the maximum abundance principle. For methods capable of outputting cell type abundance estimates, spatial gene expression profiles were further reconstructed by multiplying the abundance matrix by the reference feature matrix. Classification performance was evaluated primarily using metrics such as accuracy, weighted precision, recall, F1 score, Cohen kappa coefficient, Matthews correlation coefficient (MCC), adjusted Rand index (ARI), and normalized mutual information (NMI). All analyses were performed independently for each validation dataset within a unified computational environment, and the performance results of each algorithm were summarized and compared on a per-sample basis.

### 4.7. Trajectory Inference and Evolutionary Dynamics Analysis

The scvelo module (v0.3.2) [47] in Python was used to process the loom file output by velocyto. We imported sample metadata from the Seurat object and calculated RNA splicing and unspliced rates based on the dynamic modeling algorithm to construct cellular evolutionary trajectory models. We restored the latent dynamic process for each spot using the scv.tl.latent_time function, which reflects the spot’s intrinsic biological clock. Finally, we imported the dynamic rate data into the Dynamo module (v1.4.1) [48] to learn vector field changes and infer the evolutionary fate trajectory for each spot.

### 4.8. Combining Drug Sensitivity Analysis with In-Silico Perturbation Algorithms to Predict Potential Drug Candidates

Drug-response data, including area above the dose–response curve (AAC) and half-maximal inhibitory concentration (IC50), were obtained from the CTRP, GDSC, PRISM, CCLE, and gCSI databases using PharmacoGx (v3.6.0) [49]. AAC represents the area above the fitted dose–response curve, with higher values indicating greater drug sensitivity within the tested concentration range. IC50 represents the concentration required to reduce the measured viability-related response by 50%, with lower values generally indicating greater drug sensitivity. Compounds with available AAC or IC50 data in HCC cell lines were queried against DGIdb (v5.0) [50] to retrieve annotated drug–target associations and regulatory information. Drug targets were compared with genes represented in the inferred LE-associated transcriptional network. This analysis was used to identify potential pharmacological connections to LE-associated programs; it did not establish direct target engagement or direct inhibition of SPARC, IGFBP7, or other inferred signaling components. By Dynamo software, we performed perturbation simulations for each spot in the space: based on the dyn.pd.perturbation function, we estimated the trends in RNA velocity vector fields and state transition probabilities within the spatial region. If a drug effectively intervened in the evolutionary trajectory of the region, it was considered a preliminary candidate drug. To enhance screening reliability, we calculated drug sensitivity levels based on ST spatial expression profiles using the R package oncoPredict (v1.2) [51]. Candidates exhibiting significant sensitivity toward the LE region were prioritized by applying thresholds (|logFC| > 1 and *p*-value < 0.05). Finally, we integrated drug properties from CTD [52], SIDER [53], DrugBank [54], chEMBL [55], and PubChem [56] to prioritize treatment candidates for HCC (including assumed scores, side effects, and therapeutic feasibility). In addition, we collected drug IC50 matrices for HCC cell lines before and after gene knockout from the DepMap database [57]. We used the ggboxplot and ggscatter functions to generate box plots showing the half-maximal inhibitory concentration data for predicted candidate drugs in HCC cell lines before and after gene knockout, as well as scatter plots illustrating their correlation with gene expression.

### 4.9. Function and Pathway Analysis

We retrieved all human Hallmark gene sets (v2023.1) from MSigDB and performed gene set variation analysis (GSVA, v1.50.5) to assess differential expression and functional enrichment, screening significant functional pathways using thresholds (*p*-value < 0.01 and q-value < 0.01). To understand the structural characteristics of HCC progression, we analyzed the pathway in three histological regions using clusterProfiler (v4.10.1) [58] and calculated the LE and TC score of each sample between samples using singscore (v1.22.0) [59]. We set up different groups according to the optimal score thresholds. Visualization of results was performed using ggplot2 (v3.5.1) to represent enrichment levels of relevant pathways.

### 4.10. Analysis of Intercellular Communication Pathways

To investigate the intercellular molecular communication within the TIME and tumor structures, we have taken significantly molecular communication scores to mine significantly activated molecular signaling pathways across minimal unit level of subtypes and structures with the R package Cellchat (v2.1.2) [60]. It showed the complex communication among them, demonstrating the heterogeneity between ligand-receptor pairs and signaling gene expression patterns. Ligand–receptor interaction of molecular signaling networks has been visualized using netVisual_circle and netVisual_chord_gene. Additionally, we mapped intercellular communication networks at the spatial level to investigate molecular signaling patterns across different spatial structures.

### 4.11. Statistical Analysis

All statistical analyses were performed using R (v4.3.2) and Python (v3.12). SpaPred was implemented in Python 3.12 using PyTorch 2.13.0, scikit-learn, XGBoost, scikit-optimize, NumPy, and pandas. Random seeds were fixed at 110 for NumPy and PyTorch. Training was performed using a CUDA-enabled GPU when available; otherwise, CPU computation was used. For comparisons of continuous and categorical variables, the Mann-Whitney U test was applied for two-group comparisons, while the Kruskal–Wallis test was used for multiple-group comparisons. For normally distributed continuous variables, independent two-sample t-tests were employed. Survival differences between groups were evaluated using Kaplan–Meier analysis with the log-rank test. To control for false positives arising from multiple hypothesis testing, the Benjamini-Hochberg method was applied to adjust *p*-values, and the corresponding false discovery rate (FDR) values were reported where appropriate. A two-sided *p*-value < 0.05 (or FDR < 0.05 when applicable) was considered statistically significant.

## Data Availability

The original contributions presented in this study are included in the article/Supplementary Material. Further inquiries can be directed to the corresponding author.

## Supplementary Materials

The following supporting information can be downloaded at: https://www.mdpi.com/article/10.3390/ijms27156643/s1.
