# Supplementary material for: Deciphering the Leading-Edge Spatiotemporal Microenvironment of Hepatocellular Carcinoma for Targeted Drug Discovery Using SpaPred
Source: Int J Mol Sci. 2026 Jul 25;27(15):6643. doi: 10.3390/ijms27156643 (PMC13466823; doi:10.3390/ijms27156643)
Supplement: Supplementary file 1 [file ijms-27-06643-s001.zip › ijms-4447932-supplementary.pdf]

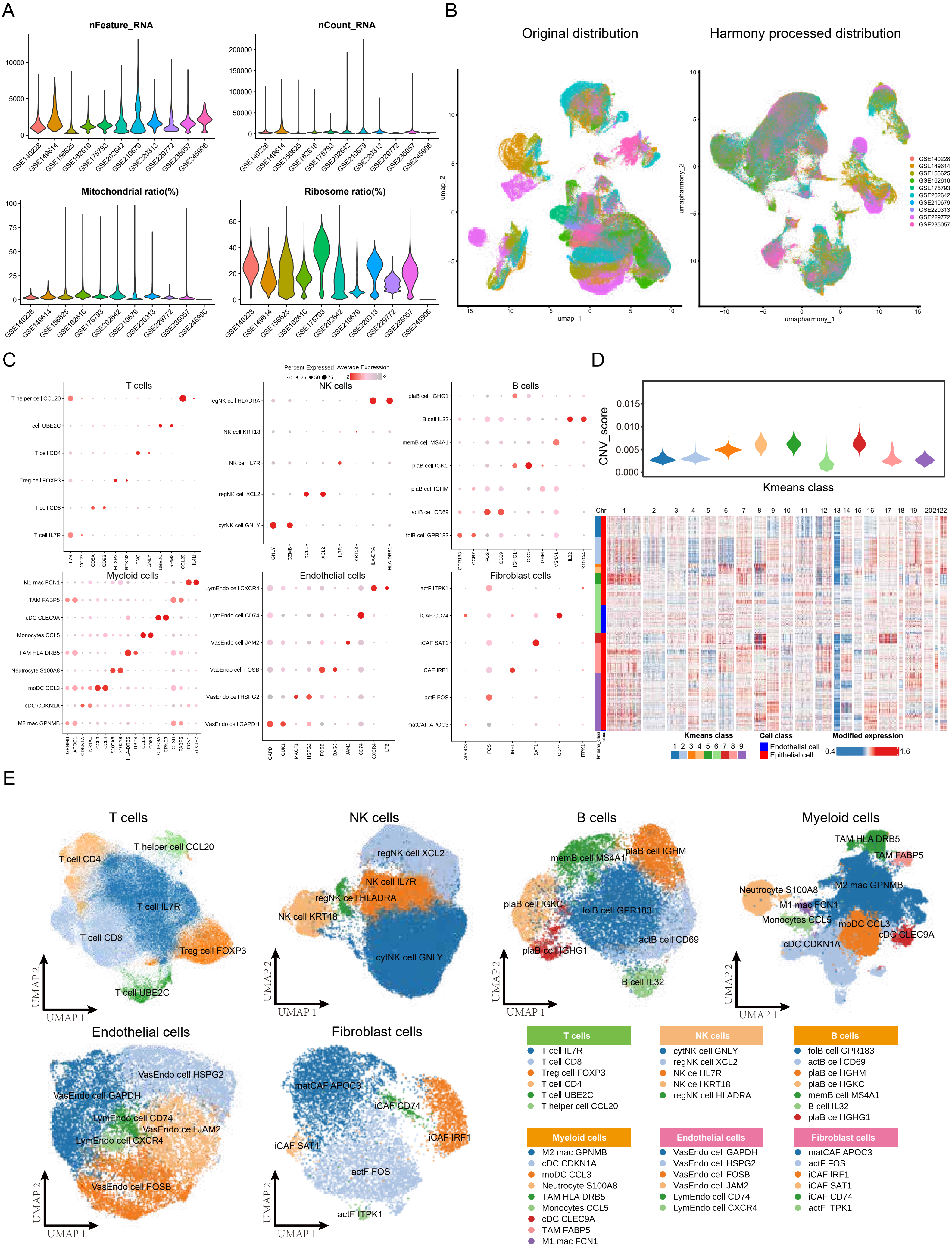

A
nFeature_RNA
nCount_RNA
Mitochondrial ratio(%)
Ribosome ratio(%)
GSE140228
GSE149614
GSE156625
GSE162616
GSE175793
GSE202642
GSE210679
GSE220313
GSE229772
GSE235057
GSE245906
B
Original distribution
Harmony processed distribution
umap_1
umap_2
umapharmony_1
umapharmony_2
C
T cells
NK cells
B cells
Myeloid cells
Endothelial cells
Fibroblast cells
Percent Expressed
Average Expression
D
CNV_score
Kmeans class
Chr
Cell class
Endothelial cell
Epithelial cell
Modified expression
E
T cells
NK cells
B cells
Myeloid cells
Endothelial cells
Fibroblast cells
UMAP 1
UMAP 2
T cells
T cell IL7R
T cell CD8
Treg cell FOXP3
T cell CD4
T cell UBE2C
T helper cell CCL20
NK cells
cytNK cell GNLY
regNK cell XCL2
NK cell IL7R
NK cell KRT18
regNK cell HLADRA
B cells
folB cell GPR183
actB cell CD69
plaB cell IGHM
plaB cell IGKC
memB cell MS4A1
B cell IL32
plaB cell IGHG1
Myeloid cells
M2 mac GPNMB
cDC CDKN1A
moDC CCL3
Neutrocyte S100A8
TAM HLA DRB5
Monocytes CCL5
cDC CLEC9A
TAM FABP5
M1 mac FCN1
Endothelial cells
VasEndo cell GAPDH
VasEndo cell HSPG2
VasEndo cell FOSB
VasEndo cell JAM2
LymEndo cell CD74
LymEndo cell CXCR4
Fibroblast cells
matCAF APOC3
actF FOS
iCAF IRF1
iCAF SAT1
iCAF CD74
actF ITPK1

Figure S1. Cell processing for single-cell transcriptomics data, related to Figure 1.

(A) Quality control metrics. (B) UMAP plots before and after Harmony algorithm processing. (C) A bubble plot showing the expression levels of various cellular markers. (D) Copy number variation scores for each k-means cluster. (E) Results of secondary clustering analysis for each cell subtype.

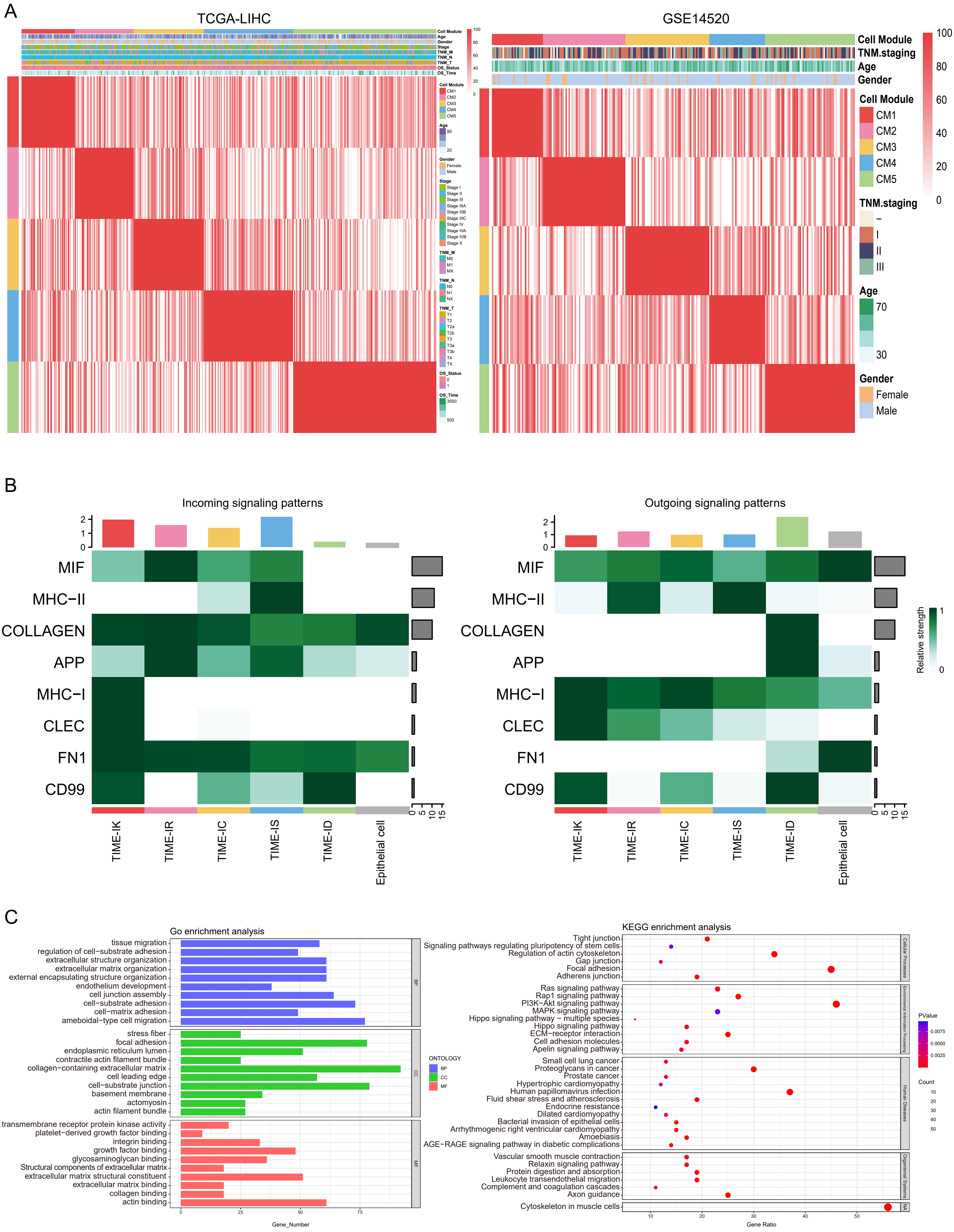
A
TCGA-LIHC
GSE14520
Cell Module
TNM.staging
Age
Gender
CM1
CM2
CM3
CM4
CM5
–
I
II
III
70
30
Female
Male
100
80
60
40
20
0
B
Incoming signaling patterns
Outgoing signaling patterns
MIF
MHC−II
COLLAGEN
APP
MHC−I
CLEC
FN1
CD99
TIME-IK
TIME-IR
TIME-IC
TIME-IS
TIME-ID
Epithelial cell
Relative strength
C
Go enrichment analysis
tissue migration
regulation of cell−substrate adhesion
extracellular structure organization
extracellular matrix organization
external encapsulating structure organization
endothelium development
cell junction assembly
cell−substrate adhesion
cell−matrix adhesion
ameboidal−type cell migration
stress fiber
focal adhesion
endoplasmic reticulum lumen
contractile actin filament bundle
collagen−containing extracellular matrix
cell leading edge
cell−substrate junction
basement membrane
actomyosin
actin filament bundle
transmembrane receptor protein kinase activity
platelet−derived growth factor binding
integrin binding
growth factor binding
glycosaminoglycan binding
Structural components of extracellular matrix
extracellular matrix structural constituent
extracellular matrix binding
collagen binding
actin binding
Gene_Number
ONTOLOGY
BP
CC
MF
KEGG enrichment analysis
Tight junction
Signaling pathways regulating pluripotency of stem cells
Regulation of actin cytoskeleton
Gap junction
Focal adhesion
Adherens junction
Ras signaling pathway
Rap1 signaling pathway
PI3K−Akt signaling pathway
MAPK signaling pathway
Hippo signaling pathway − multiple species
Hippo signaling pathway
ECM−receptor interaction
Cell adhesion molecules
Apelin signaling pathway
Small cell lung cancer
Proteoglycans in cancer
Prostate cancer
Hypertrophic cardiomyopathy
Human papillomavirus infection
Fluid shear stress and atherosclerosis
Endocrine resistance
Dilated cardiomyopathy
Bacterial invasion of epithelial cells
Arrhythmogenic right ventricular cardiomyopathy
Amoebiasis
AGE−RAGE signaling pathway in diabetic complications
Vascular smooth muscle contraction
Relaxin signaling pathway
Protein digestion and absorption
Leukocyte transendothelial migration
Complement and coagulation cascades
Axon guidance
Cytoskeleton in muscle cells
Cellular Processes
Human Diseases
Organismal Systems
NA
Gene Ratio
PValue
Count

Figure S2. Analysis of signaling pathways and functions in the TIME subtype, related to Figure 2.

(A) Results of cellular module enrichment across the sample population in the TCGA-LIHC cohort and the GSE14520 dataset. (B) Heatmap showing the weights of input and output signaling molecule patterns between the TIME subtype and malignant epithelial cells. (C) GO and KEGG functional enrichment analysis of the TIME-ID phenotype.

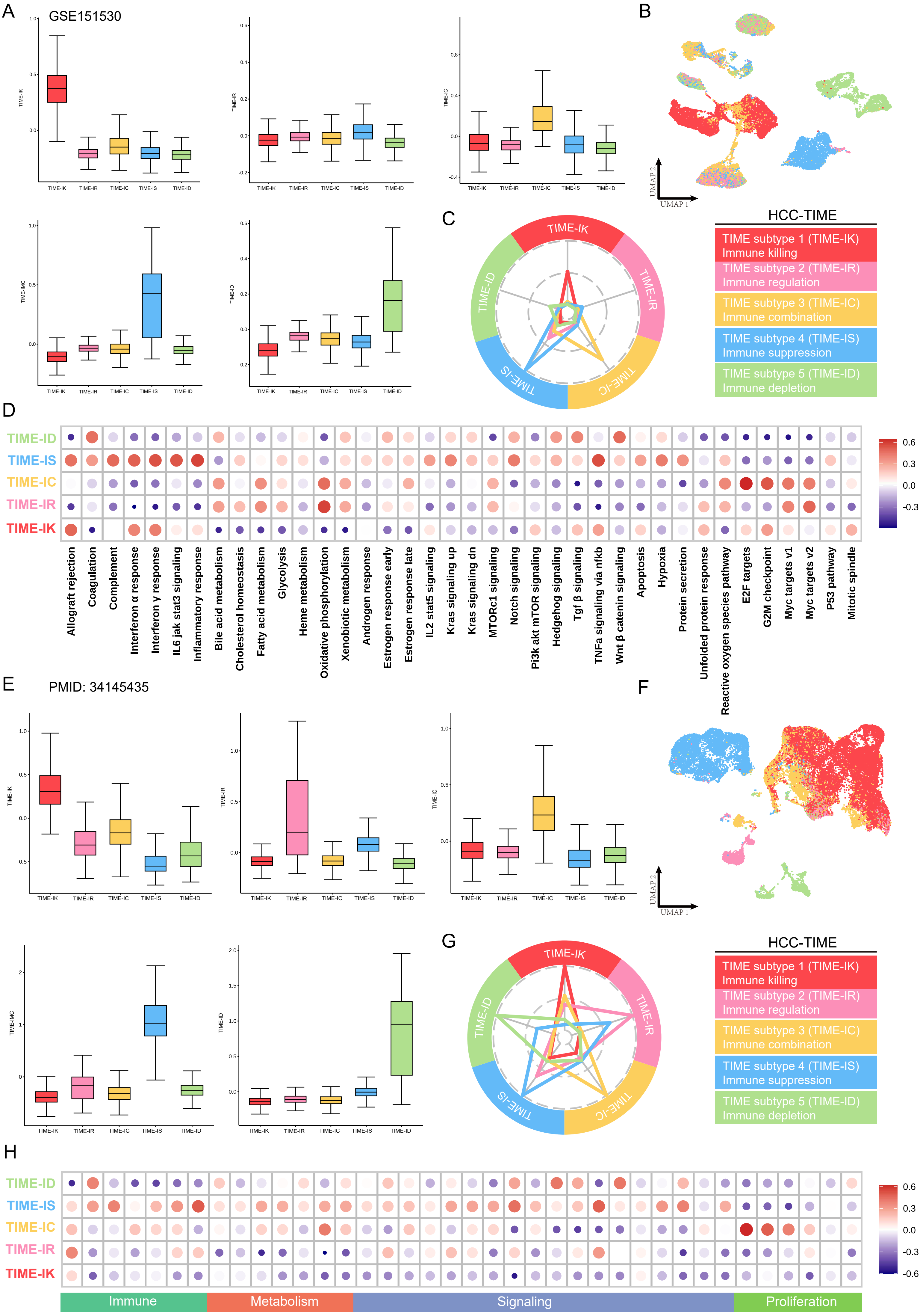

A
GSE151530
TIME-IK
TIME-IR
TIME-IC
TIME-IS
TIME-ID
TIME-IMC
B
UMAP 2
UMAP 1
C
HCC-TIME
TIME subtype 1 (TIME-IK) Immune killing
TIME subtype 2 (TIME-IR) Immune regulation
TIME subtype 3 (TIME-IC) Immune combination
TIME subtype 4 (TIME-IS) Immune suppression
TIME subtype 5 (TIME-ID) Immune depletion
D
Allograft rejection
Coagulation
Complement
Interferon α response
Interferon γ response
IL6 jak stat3 signaling
Inflammatory response
Bile acid metabolism
Cholesterol homeostasis
Fatty acid metabolism
Glycolysis
Heme metabolism
Oxidative phosphorylation
Xenobiotic metabolism
Androgen response
Estrogen response early
Estrogen response late
IL2 stat5 signaling
Kras signaling up
Kras signaling dn
MTORc1 signaling
Notch signaling
Pi3k akt mTOR signaling
Hedgehog signaling
Tgf β signaling
TNFa signaling via nfkb
Wnt β catenin signaling
Apoptosis
Hypoxia
Protein secretion
Unfolded protein response
Reactive oxygen species pathway
E2F targets
G2M checkpoint
Myc targets v1
Myc targets v2
P53 pathway
Mitotic spindle
0.6
0.3
0.0
-0.3
E
PMID: 34145435
F
G
H
-0.6
Immune
Metabolism
Signaling
Proliferation

Figure S3. External datasets validate the accuracy of TIME subtyping, related to Figure 2.
(A)Box plots demonstrate the enrichment results for TIME isoforms in GSE151530. (B) Distribution of TIME subtypes in the 2D plane after UMAP downscaling. (C) Radar graph showing expression levels of TIME subtype markers. (D)Results of KEGG enrichment analysis of the TIME-ID phenotype. (E) Results of enrichment of TIME subtypes in data obtained via literature 34145435. (F) Distribution of TIME subtypes. (G) Radar chart of the TIME subtype. (H) GSVA enrichment results for each subtype of TIME.

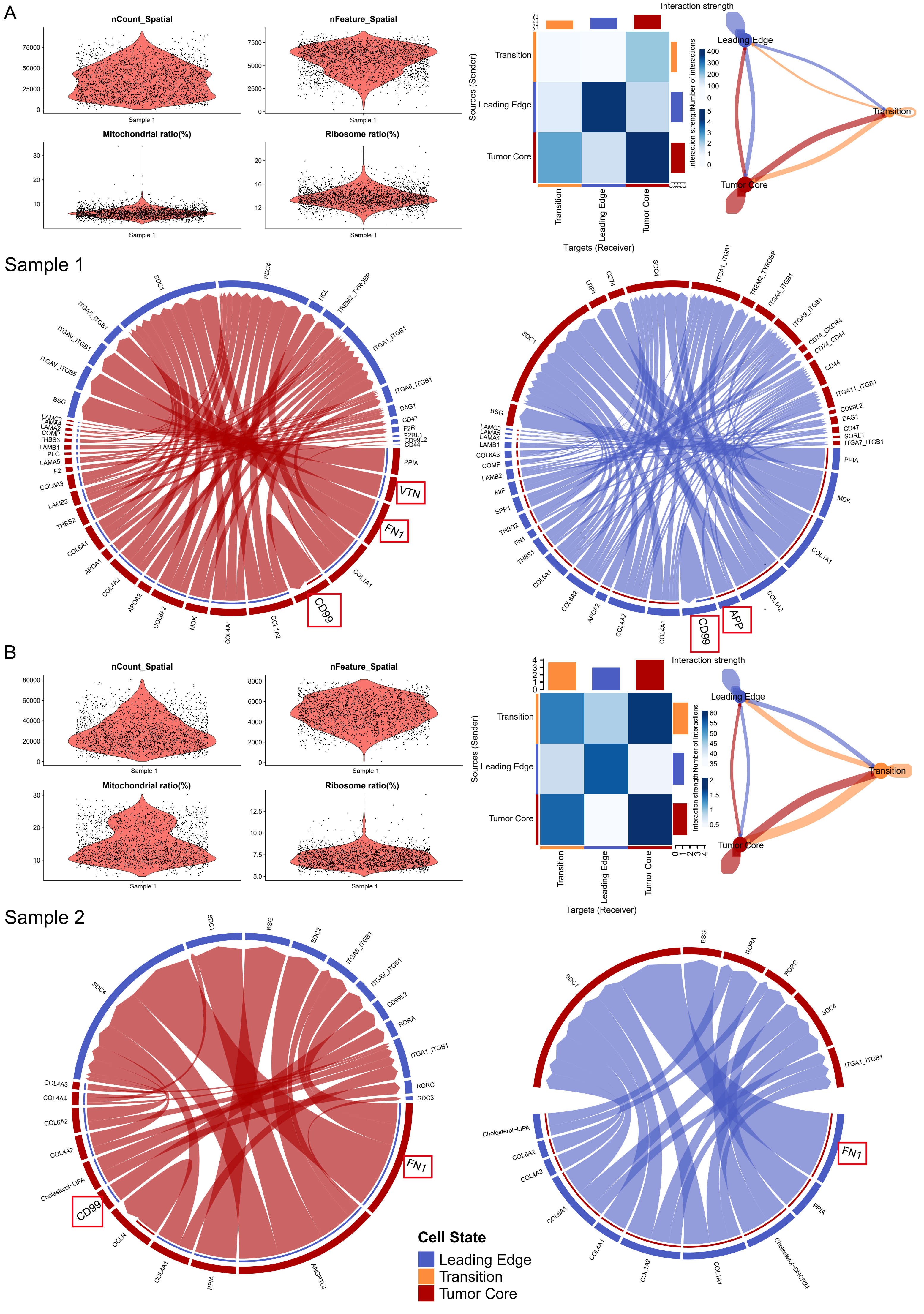
A
nCount_Spatial
nFeature_Spatial
Mitochondrial ratio(%)
Ribosome ratio(%)
Sample 1
Interaction strength
Sources (Sender)
Targets (Receiver)
Transition
Leading Edge
Tumor Core
Number of interactions
Interaction strength
Sample 1
SDC4
SDC1
ITGA5_ITGB1
ITGAV_ITGB1
ITGAV_ITGB5
BSG
NCL
TREM2_TYROBP
ITGA1_ITGB1
ITGA6_ITGB1
DAG1
CD47
F2R
F2RL1
CD99L2
CD44
PPIA
VTN
FN1
COL1A1
CD99
COL1A2
COL4A1
MDK
COL6A2
APOA2
COL4A2
APOA1
COL6A1
THBS2
LAMB2
COL6A3
F2
LAMA5
PLG
LAMB1
THBS3
COMP
LAMA2
LAMA4
LAMC3
LRP1
CD74
ITGA4_ITGB1
ITGA9_ITGB1
CD74_CXCR4
CD74_CD44
ITGA11_ITGB1
SORL1
ITGA7_ITGB1
MDK
MIF
SPP1
THBS1
APP
B
Sample 2
SDC2
CD99L2
RORA
RORC
SDC3
COL4A3
COL4A4
Cholesterol−LIPA
OCLN
ANGPTL4
Cholesterol−DHCR24
Cell State
Leading Edge
Transition
Tumor Core

Figure S4. Quality control and functional analysis of spatial transcriptomics data, related to Figure 3.

(A) Quality control metrics for spatial transcriptomics sample 1, signal levels among spatial structures within the tumor, signal propagation within TCs and LEs, and the enrichment of tumor microenvironment features in tumor spatial structures. (B) Quality control metrics for spatial transcriptomics sample 2, signal levels among spatial structures within the tumor, signal propagation within TCs and LEs, and the enrichment of tumor microenvironment features in tumor spatial structures.

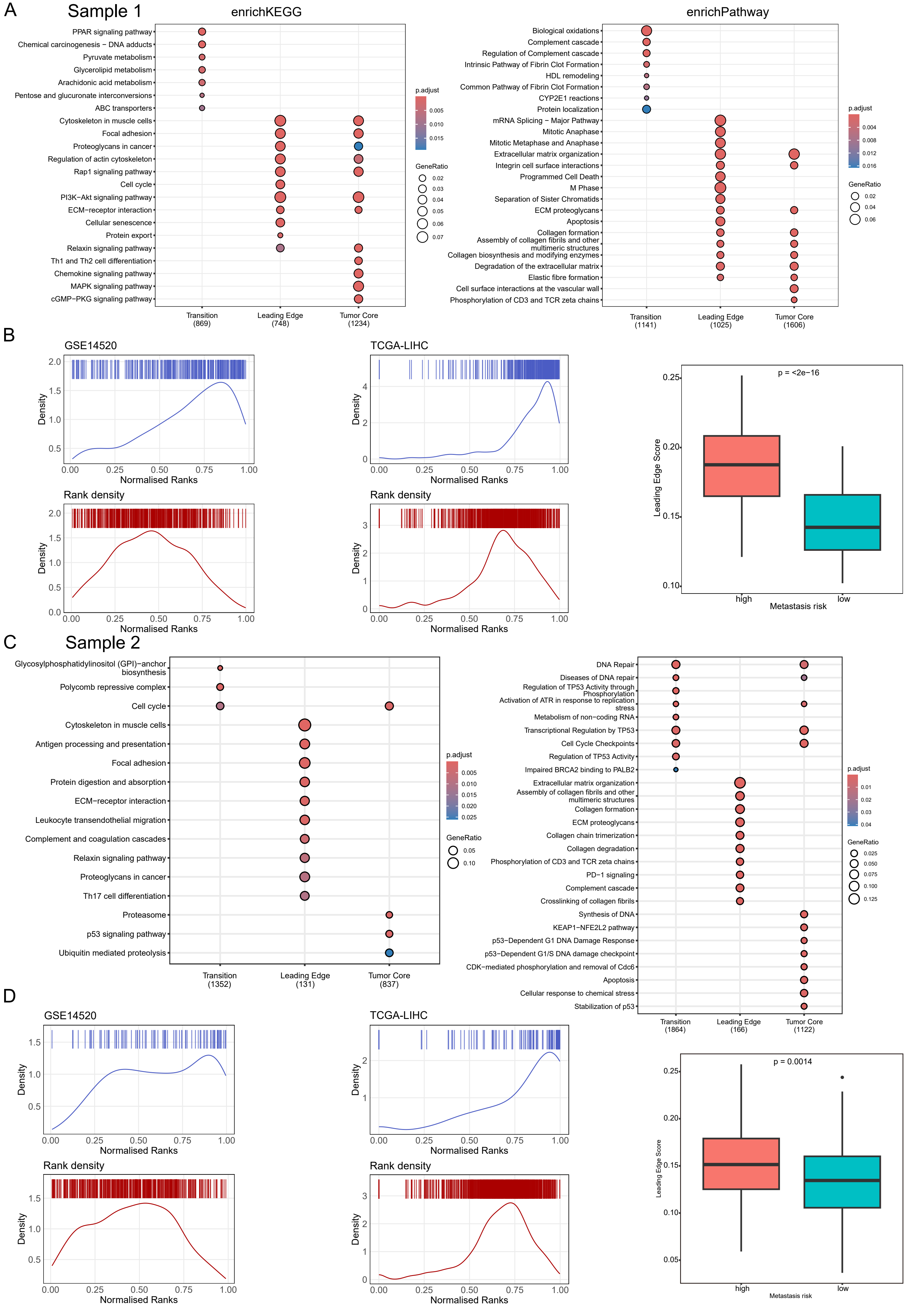

A
Sample 1
enrichKEGG
PPAR signaling pathway
Chemical carcinogenesis − DNA adducts
Pyruvate metabolism
Glycerolipid metabolism
Arachidonic acid metabolism
Pentose and glucuronate interconversions
ABC transporters
Cytoskeleton in muscle cells
Focal adhesion
Proteoglycans in cancer
Regulation of actin cytoskeleton
Rap1 signaling pathway
Cell cycle
PI3K−Akt signaling pathway
ECM−receptor interaction
Cellular senescence
Protein export
Relaxin signaling pathway
Th1 and Th2 cell differentiation
Chemokine signaling pathway
MAPK signaling pathway
cGMP−PKG signaling pathway
Transition (869)
Leading Edge (748)
Tumor Core (1234)
p.adjust
0.005
0.010
0.015
GeneRatio
0.02
0.03
0.04
0.05
0.06
0.07
enrichPathway
Biological oxidations
Complement cascade
Regulation of Complement cascade
Intrinsic Pathway of Fibrin Clot Formation
HDL remodeling
Common Pathway of Fibrin Clot Formation
CYP2E1 reactions
Protein localization
mRNA Splicing − Major Pathway
Mitotic Anaphase
Mitotic Metaphase and Anaphase
Extracellular matrix organization
Integrin cell surface interactions
Programmed Cell Death
M Phase
Separation of Sister Chromatids
ECM proteoglycans
Apoptosis
Collagen formation
Assembly of collagen fibrils and other multimeric structures
Collagen biosynthesis and modifying enzymes
Degradation of the extracellular matrix
Elastic fibre formation
Cell surface interactions at the vascular wall
Phosphorylation of CD3 and TCR zeta chains
Transition (1141)
Leading Edge (1025)
Tumor Core (1606)
p.adjust
0.004
0.008
0.012
0.016
GeneRatio
0.02
0.04
0.06
B
GSE14520
TCGA-LIHC
Density
Normalised Ranks
Rank density
p = <2e−16
Leading Edge Score
high
low
Metastasis risk
C
Sample 2
Glycosylphosphatidylinositol (GPI)−anchor biosynthesis
Polycomb repressive complex
Cell cycle
Cytoskeleton in muscle cells
Antigen processing and presentation
Focal adhesion
Protein digestion and absorption
ECM−receptor interaction
Leukocyte transendothelial migration
Complement and coagulation cascades
Relaxin signaling pathway
Proteoglycans in cancer
Th17 cell differentiation
Proteasome
p53 signaling pathway
Ubiquitin mediated proteolysis
Transition (1352)
Leading Edge (131)
Tumor Core (837)
p.adjust
0.005
0.010
0.015
0.020
0.025
GeneRatio
0.05
0.10
DNA Repair
Diseases of DNA repair
Regulation of TP53 Activity through Phosphorylation
Activation of ATR in response to replication stress
Metabolism of non−coding RNA
Transcriptional Regulation by TP53
Cell Cycle Checkpoints
Regulation of TP53 Activity
Impaired BRCA2 binding to PALB2
Extracellular matrix organization
Assembly of collagen fibrils and other multimeric structures
Collagen formation
ECM proteoglycans
Collagen chain trimerization
Collagen degradation
Phosphorylation of CD3 and TCR zeta chains
PD−1 signaling
Complement cascade
Crosslinking of collagen fibrils
Synthesis of DNA
KEAP1−NFE2L2 pathway
p53−Dependent G1 DNA Damage Response
p53−Dependent G1/S DNA damage checkpoint
CDK−mediated phosphorylation and removal of Cdc6
Apoptosis
Cellular response to chemical stress
Stabilization of p53
Transition (1864)
Leading Edge (166)
Tumor Core (1122)
p.adjust
0.01
0.02
0.03
0.04
GeneRatio
0.025
0.050
0.075
0.100
0.125
D
GSE14520
TCGA-LIHC
Density
Normalised Ranks
Rank density
p = 0.0014
Leading Edge Score
high
low
Metastasis risk

Figure S5. Functional enrichment analysis of tumor spatial structures, related to Figure 3.
(A) A bubble plot showing KEGG and pathway enrichment results for different tumor spatial regions in spatial transcriptomics sample 1. (B) GSEA distribution results for LE scores and TC scores in spatial transcriptomics sample 1, as well as differences in metastasis trends and prognosis among patients grouped by LE scores. (C) A bubble plot showing KEGG and pathway enrichment results for different tumor spatial regions in Spatial Transcriptome Sample 2. (D) GSEA distribution results for LE scores and TC scores in Spatial Transcriptome Sample 2, as well as differences in metastasis trends and prognosis among patients grouped by LE scores.

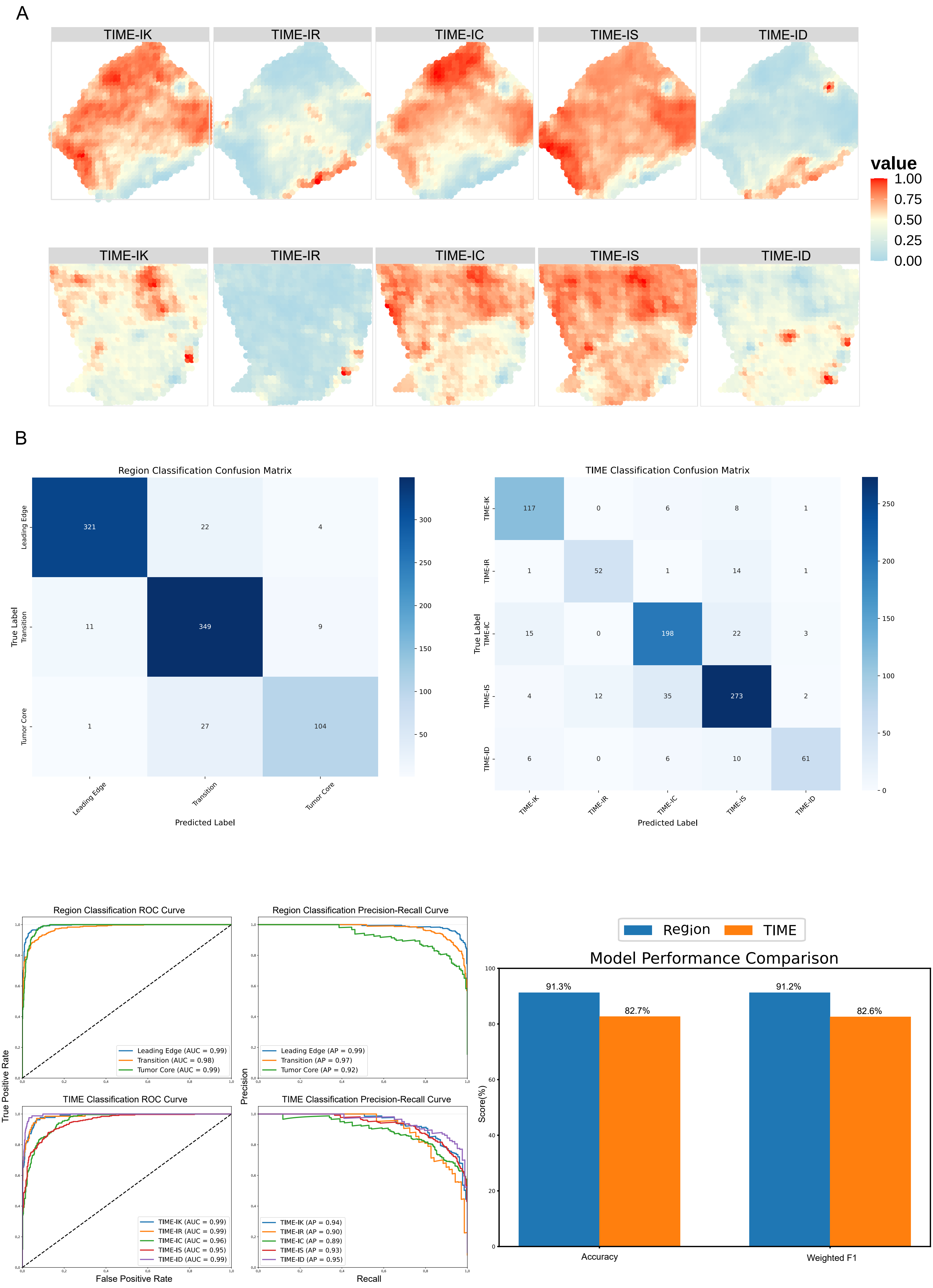

A
TIME-IK
TIME-IR
TIME-IC
TIME-IS
TIME-ID
value
1.00
0.75
0.50
0.25
0.00
B
Region Classification Confusion Matrix
True Label
Predicted Label
Leading Edge
Transition
Tumor Core
321
22
4
11
349
9
1
27
104
TIME Classification Confusion Matrix
117
0
6
8
1
1
52
1
14
1
15
0
198
22
3
4
12
35
273
2
6
0
6
10
61
Region Classification ROC Curve
Leading Edge (AUC = 0.99)
Transition (AUC = 0.98)
Tumor Core (AUC = 0.99)
Region Classification Precision-Recall Curve
Leading Edge (AP = 0.99)
Transition (AP = 0.97)
Tumor Core (AP = 0.92)
TIME Classification ROC Curve
TIME-IK (AUC = 0.99)
TIME-IR (AUC = 0.99)
TIME-IC (AUC = 0.96)
TIME-IS (AUC = 0.95)
TIME-ID (AUC = 0.99)
TIME Classification Precision-Recall Curve
TIME-IK (AP = 0.94)
TIME-IR (AP = 0.90)
TIME-IC (AP = 0.89)
TIME-IS (AP = 0.93)
TIME-ID (AP = 0.95)
True Positive Rate
False Positive Rate
Precision
Recall
Region
TIME
Model Performance Comparison
Score(%)
91.3%
82.7%
91.2%
82.6%
Accuracy
Weighted F1

Figure S6. Spatial deconvolution of tumor TIME subtypes and performance analysis of the SpaPred model, related to Figure 5.

(A) Distribution of spatial mappings of tumor TIME subtypes across different samples. (B) Confusion matrix of the SpaPred model's predictions, performance and generalization curves, and the model's prediction accuracy versus F1 score.

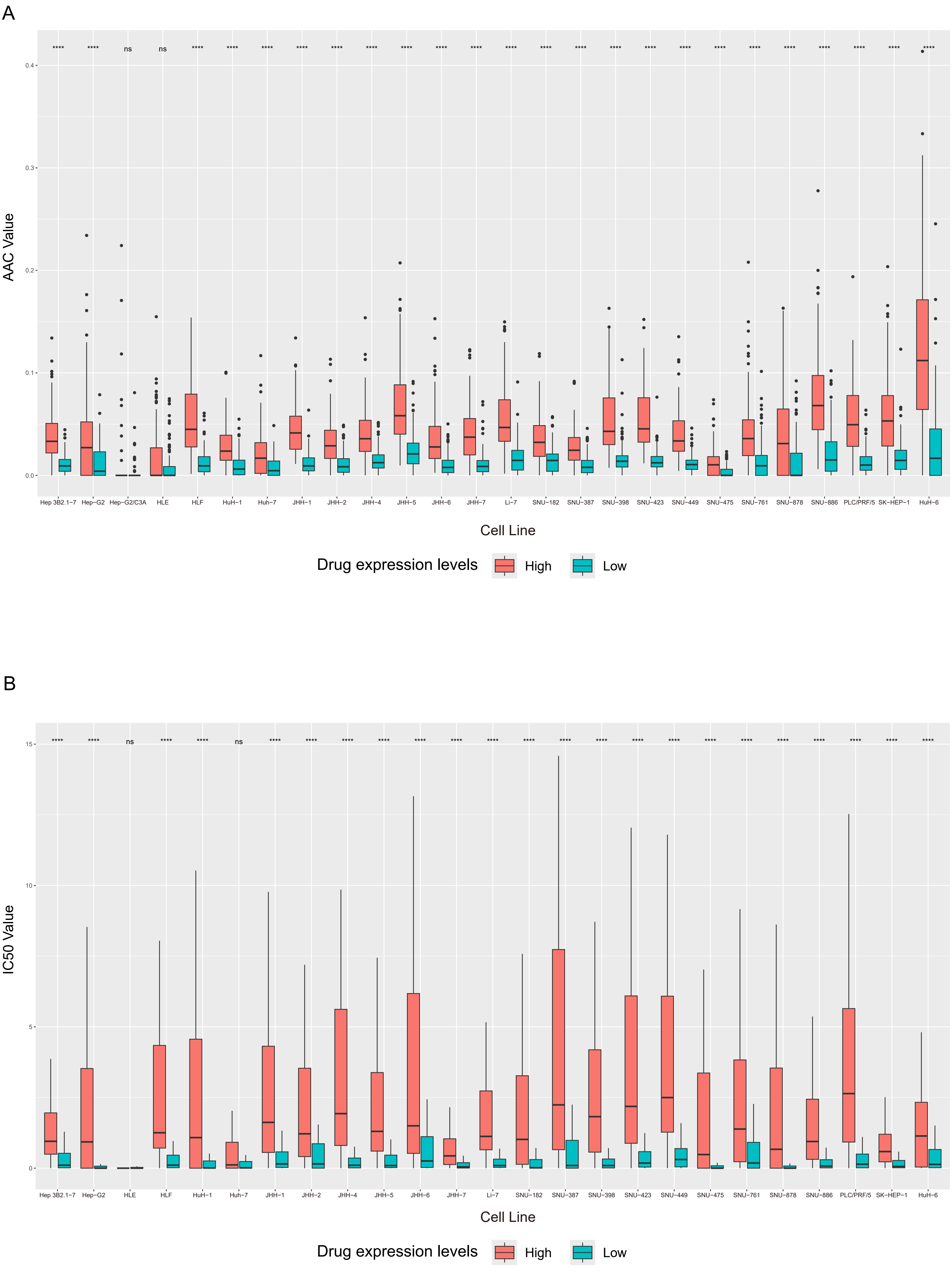
A
AAC Value
0.0
0.1
0.2
0.3
0.4
Hep 3B2.1−7
Hep−G2
Hep−G2/C3A
HLE
HLF
HuH−1
Huh−7
JHH−1
JHH−2
JHH−4
JHH−5
JHH−6
JHH−7
Li−7
SNU−182
SNU−387
SNU−398
SNU−423
SNU−449
SNU−475
SNU−761
SNU−878
SNU−886
PLC/PRF/5
SK−HEP−1
HuH−6
Cell Line
Drug expression levels
High
Low
B
IC50 Value
0
5
10
15
Hep 3B2.1−7
Hep−G2
HLE
HLF
HuH−1
Huh−7
JHH−1
JHH−2
JHH−4
JHH−5
JHH−6
JHH−7
Li−7
SNU−182
SNU−387
SNU−398
SNU−423
SNU−449
SNU−475
SNU−761
SNU−878
SNU−886
PLC/PRF/5
SK−HEP−1
HuH−6
Cell Line
Drug expression levels
High
Low

Figure S7. Expression levels of AAC and IC50 in drug-sensitive cell lines, related to Figure 6. (A) Expression levels of 26 HCC cell lines across two groups in the AAC score. (B) Expression levels of 26 HCC cell lines across two groups in the IC50 score.

Table S1. Gene signatures developed for the TIME classification

Checkpoint molecules	Treg	Co-activation molecules	Neutrophil signature	Immune Suppression by Myeloid Cells	Pro-tumor cytokines	Matrix	Cancer-associated fibroblasts	Endothelium
PDCD1	FOXP3	CD28	MPO	IDO1	IL10	FN1	COL1A1	NOS3
CD274	CTLA4	CD40	ELANE	ARG1	TGFB1	COL1A1	COL1A2	KDR
CTLA4	IL10	TNFRSF4	PRTN3	IL10	TGFB2	COL1A2	COL5A1	FLT1
LAG3	TNFRSF18	ICOS	CTSG	CYBB	TGFB3	COL4A1	ACTA2	VCAM1
PDCD1LG2	CCR8	TNFRSF9	CXCR1	PTGS2	IL22	COL3A1	FGF2	VWF
BTLA	IKZF4	CD27	CXCR2	IL4I1	MIF	VTN	FAP	CDH5
HAVCR2	IKZF2	CD80	FCGR3B	IL6	IL6	LGALS7	LRP1	MMRN1
TIGIT		CD86	CD177			LGALS9	CD248	ENG
VSIR		CD40LG	FFAR2			LAMA3	COL6A1	CLEC14A
C10orf54		CD83	PGLYRP1			LAMB3	COL6A2	MMRN2
		TNFSF4				LAMC2	COL6A3	
		ICOSLG				TNC	CXCL12	
		TNFSF9				ELN	FBLN1	
		CD70				COL5A1	LUM	
						COL11A1	MFAP5	
							MMP3	
							MMP2	
							PDGFRB	
							PDGFRA	

Angiogenesis	T cells	Effector cells
VEGFA	TBX21	IFNG
VEGFB	ITK	GZMA
VEGFC	CD3D	GZMB
PDGFC	CD3E	PRF1
CXCL8	CD3G	GZMK
CXCR2	TRAC	ZAP70
FLT1	TRBC1	GNLY
PGF	TRBC2	FASLG
CXCL5	CD28	TBX21
KDR	CD5	EOMES
ANGPT1	TRAT1	CD8A
ANGPT2		CD8B
TEK		
VWF		
CDH5		

Table S2. Detailed information on datasets used in this study.						
Category	Dataset	Source	Platform	Disease	Samples	Cells/Spots QC After
scRNA	GSE140228	GEO	10x Genomics	HCC	12	16711 Cells
scRNA	GSE149614	GEO	10x Genomics	HCC	10	20128 Cells
scRNA	GSE156625	GEO	10x Genomics	HCC	57	43008 Cells
scRNA	GSE162616	GEO	10x Genomics	HCC	3	37099 Cells
scRNA	GSE175793	GEO	10x Genomics	HCC	1	12266 Cells
scRNA	GSE202642	GEO	10x Genomics	HCC	7	41500 Cells
scRNA	GSE210679	GEO	10x Genomics	HCC	1	4197 Cells
scRNA	GSE220313	GEO	10x Genomics	HCC	2	9053 Cells
scRNA	GSE224411	GEO	10x Genomics	HCC	1	937 Cells
scRNA	GSE229772	GEO	10x Genomics	HCC	27	46020 Cells
scRNA	GSE235057	GEO	10x Genomics	HCC	10	43825 Cells
scRNA	GSE245906	GEO	10x Genomics	HCC	10	56229 Cells
scRNA-Val	GSE151530	GEO	10x Genomics	HCC	32	27391 Cells
scRNA-Val	PMID34145435	NA	NA	HCC	6	83793 Cells
ST	GSE245908	GEO	10X Visium	HCC	2	2261 Spots
ST-Val	HRA000437	CNCB	10X Visium	HCC	8	1987 Spots
Bulk	TCGA-LIHC	TCGA	RNA-seq	LIHC	418	NA
Bulk	GSE14520	GEO	RNA-seq	HCC	242	NA
